# Pharmacological Therapies for Consequences of Perinatal Hypoxic-Ischemic Brain Injury: Where Are We Now?

**DOI:** 10.3390/ijms262010200

**Published:** 2025-10-20

**Authors:** Paulina Gebala, Justyna Janowska, Joanna Sypecka

**Affiliations:** 1 NeuroRepair Department, Mossakowski Medical Research Institute, Polish Academy of Sciences, Pawinskiego 5, 02-106 Warsaw, Poland; pgebala@imdik.pan.pl (P.G.); jjanowska@imdik.pan.pl (J.J.); 2 Centre of Postgraduate Medical Education, Doctoral School of Translational Medicine, Marymoncka 99/103, 01-813 Warsaw, Poland

**Keywords:** neonatal brain, hypoxic-ischemic injury, neuroprotection, neuroregeneration, pharmacological therapies, medicinal chemistry

## Abstract

Despite significant progress in preclinical research aimed at developing effective therapies for the acute and long-term consequences of perinatal asphyxia, there is still a lack of clinical protocols to regenerate the neonatal brain damaged by hypoxic-ischemic (HI) injury. To date, only therapeutic hypothermia is routinely used in neonates who have experienced perinatal asphyxia. It has been shown to be effective only in limiting the spread of brain damage caused by a cascade of molecular and biochemical events triggered by limited blood supply to the body’s organs, including the fragile, developing brain. Ongoing clinical trials are exploring pharmacological approaches aimed at promoting neurogenesis and gliogenesis to repair damaged neural tissue, as well as modulating the neuroinflammation that results from the cellular response to HI injury. Among promising therapeutic agents, erythropoietin, and melatonin have emerged as major drugs with potential neuroprotective effects in neonatal hypoxic-ischemic encephalopathy. Erythropoietin is recognized for its anti-apoptotic, anti-oxidative, and anti-inflammatory properties, supporting neural cell survival and regeneration. Melatonin acts as a potent antioxidant and anti-inflammatory agent, helping to reduce oxidative stress and inflammation triggered by HI injury. As clinical trials on suffering neonates are highly demanding, the ethical and practical concerns of therapeutic approaches are discussed. An urgent need to develop a safe, feasible, and effective clinical approach to promote the restoration of appropriate neurodevelopment in the near future is highlighted. This review summarizes the clinical trials conducted to date, discusses their outcomes and limitations, and considers translational potential of the tested treatment strategies.

## 1. Introduction

Hypoxic-ischemic encephalopathy (HIE) is a neurological condition that arises in newborns due to temporally reduced blood flow leading to lowered supply in oxygen and nutrients essential for sustaining the brain’s high energy requirements. Inadequate supply of oxygen to the brain manifests as a spectrum of symptoms ranging from altered consciousness and decreased spontaneous movements, muscle tone, and reflexes to seizures in severe cases. Approximately 35% of newborns who suffered from HIE experienced long-lasting neurodevelopmental consequences, including conditions like intellectual disability, epilepsy, cerebral palsy, hearing impairment, and learning challenges. Unfortunately, up to 25% of babies who develop HIE from perinatal asphyxia do not survive beyond the first two years of life [1,2].

The signs of perinatal HI shortly after birth include low Apgar scores [3], low rates of umbilical cord gases (pH < 7.0 or base deficit ≥12 mmol/L) [4], the requirement for respiratory assistance [5,6], and abnormal fetal heart rate patterns.

Hypoxic-ischemic brain injury is pathophysiologically relevant to ischemic diseases such as myocardial infarction. This is most often caused by sudden closure of a coronary artery, which results in loss of oxygen supply to the heart muscle and necrosis of cardiomyocytes. In both cases, ischemia and reperfusion play a key role, as do the inflammatory response and cell death mechanisms. Myocardial infarction results from ischemia when it lasts too long, and it is now understood that reperfusion, although essential to terminate ischemic injury, also causes additional irreversible damage [7,8]. The same situation is in newborns suffering from severe asphyxia where reperfusion may exacerbate damage caused by free radicals. Dying and dead cardiomyocytes release and present cellular debris, then cytokines, chemokines, and various inflammatory mediators released, activating the innate immune response. The immediate reaction, facilitated by reperfusion, is driven by neutrophils [9,10,11]. This corresponds to the mechanisms of inflammation and repair after HIE—the activation of innate immunity, influx of neutrophils, and macrophages, which can cause secondary damage to the heart. One of the most important methods that has a beneficial effect on the size of the infarct is mild hypothermia, but this effect is observed more often in preclinical models than in patients. In the case of HIE, however, TH is crucial to limit the extent of the injury [12,13,14].

**Figure 1:**
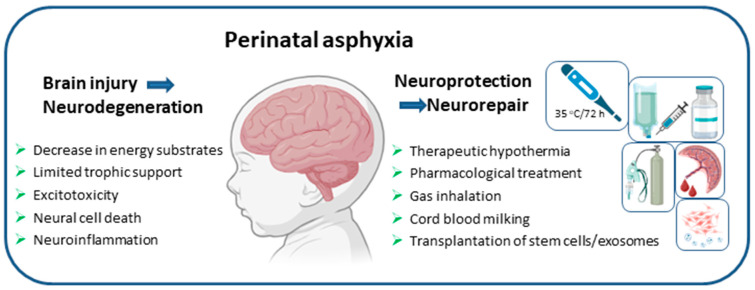
Perinatal asphyxia leads to a cascade of biochemical events that trigger the initiation of neurodegenerative processes in the fragile, developing brain. Therapeutic strategies are aimed at limiting brain damage and promoting neuroreparative processes.

Therapeutic strategies that may be able to reverse or at least mitigate the negative effects of hypoxic-ischemic injury are under intense investigation. The following sections focus on the pharmacological treatment options that have undergone or are planned to undergo clinical trials, and consider their potential for translation into neonatal clinics in the near future. Drug delivery to affected neonates could be a long-term medical strategy, supporting both neurodevelopmental processes and neurological outcomes throughout childhood (Figure 1).

## 2. Ethical and Practical Concerns of Clinical Trials in Neonates

Neonatal care in everyday practice and clinical trials in this special group of patients present many challenges. First of all, the injuries affect the brain, which is extremely fragile and still developing. The mechanisms initiated by temporal limitation of oxygen and metabolic substrates are still under investigation. Taking into consideration that the perinatal period coincides with the intense processes of neurogenesis and gliogenesis, the neural cells in their progenitor state are very vulnerable to insults that affect intercellular signal transduction pathways (such as autophagy, biosynthesis of structural and signaling elements, paracrine activity) or induce cell death [15,16,17,18,19]. Cellular targets for therapeutic strategies therefore remain to be identified.

Additionally, treatments should be based on active compounds that have a significant lifespan (are not rapidly metabolized after administration) and are able to cross the brain-blood barrier (BBB), although in the perinatal and early postnatal period, the developing BBB are still sealed by the astrocytic end feet. To date, strategies are predominantly aimed at limiting the brain injury (saving the dying neurons and glial cells), governing the neuroinflammation, and promoting (or enforcing in the later period) the endogenous neuroregenerative processes. While the local inflammatory process is thought to be beneficial for the nervous tissue, helping to clear the tissue of cellular debris left by dying cells, prolonged inflammation may hinder neurorestoration of the nervous tissue cytoarchitecture. Thus, the application of mild anti-inflammatory compounds or the use of stem cell secretomes may help to modulate the intensity of ongoing damaging processes and make the tissue microenvironment more conducive to the initiation of regenerative mechanisms [20].

However, in critically injured neonates, the outcome of treatment can be unpredictable, especially as patients usually require additional individualized treatment to support their survival and medical comfort. Unpredictable outcomes are the serious obstacles for obtaining the obligatory informed consent from the parents or legal guardians, which is necessary to enroll the affected neonate in the planned clinical trial [18]. As newborn babies are not in a position to choose to expose themselves to a certain level of risk, adults must rely heavily on the available information about the results of pre-clinical data and the medical personnel conducting the trial. It is therefore not surprising that hypothermia, which is reasonably effective and not excessively risky, is still the most commonly used treatment. However, body/head cooling, which helps to limit the extent of brain injury, needs to be supported by therapies that exert neuroprotective, antioxidant and anti-inflammatory effects, thereby promoting neuroreparative processes. A range of pharmacological compounds are being tested to verify their utility as a potential treatment for the fatal consequences of neonatal hypoxic-ischemic insult.

## 3. Erythropoietin

Erythropoietin (EPO) is a cytokine that serves various functions beyond being a hematopoietic growth factor. Its receptors are found in neurons, glial cells, and endothelial cells [21,22]. The receptors participate in the proliferation and differentiation of these cells both during normal brain development and in response to hypoxia [23,24,25,26,27]. Hypoxia and pro-inflammatory cytokines modulate activation of hypoxia-inducible factors, which leads to the expression of EPO and its receptors [28]. EPO offers neuroprotection by promoting anti-apoptotic, anti-oxidative, and anti-inflammatory effects [29,30]. Moreover, EPO enhances neuronal and glial migration around the injured site by stimulating the secretion of matrix metalloproteinases [31]. The expression of the EPO gene is primarily regulated by hypoxia. Key regulators of this gene expression include hypoxia-inducible factors (HIF) 1, 2, and 3, along with nuclear factor kappa B (NF-kB) [32,33,34]. Recent findings indicate that hypoxia-inducible factor 2a (HIF-2a) plays a significant role in regulating erythropoietin gene expression in hepatic cells [35,36].

Erythropoietin-stimulating agents (ESAs) are pharmacologically manufactured recombinant forms of EPO, created using recombinant DNA technology in cell cultures. Examples of these agents include EPO α, darbepoetin, and methoxy polyethylene glycol-epoetin β [37].

In the clinical study (NCT00719407) the researchers administered EPO α (Procrit) as a therapy for HIE in newborns. This intervention (parallel assignment) in a phase I trial tested the safety and pharmacokinetics of EPO in neonates during HIE. Each patient received intravenously six doses of Procrit at one of four available concentrations: 250 (*n* = 3), 500 (*n* = 6), 1000 (*n* = 7), and 2500 U/kg per dose (*n* = 8). Meanwhile, the neonates were cooled with the target temperature for 72 h (whole body hypothermia: *n* = 21 or cooling cap, *n* = 3). The study aimed to develop a drug dose that will result in increased neurodevelopment in children affected by HIE [38,39]. The monitoring of neurological symptoms typical for HIE included tone abnormalities, cerebral palsy, language delay, motor delay, and episodes of seizures. Although it was not possible to develop a therapeutic protocol on the basis of this study, the use of EPO, even in high doses, has been shown to have no significant side effects, but reported cases of moderate to severe disabilities (as much as 4.5%) and cases of brain injury (corresponding to 36%).

The interventional (parallel assignment) study in a phase III (NCT03079167) aimed to determine whether the combination of EPO and hypothermia in infants with moderate to severe HIE improves neurodevelopmental outcomes at 2 years of age compared to hypothermia alone, without causing significant adverse effects. The experimental group of neonates received intravenously 1000 IU/kg (capped at 4000 IU/kg per day) of EPO α (Procrit/Epogen, Amgen, Thousand Oaks, CA, USA) at 1, 2, 3, 5, and 7 days of age. The placebo group received 0.9% normal saline intravenously at the same intervals. Whole-body hypothermia was also administered to both groups in the trial. The trial was completed at the end of April 2024, but the results have not yet been published or posted on the clinical trial website.

The results of another study using EPO are also not available. It was the interventional (parallel assignment) in a phase I and II (NCT00808704) study, in which the recombinant human EPO (r-hu-EPO) was administered in doses corresponding to either 300 U/kg or 500 U/kg. The drug was first given subcutaneously, then intravenously every day for 2 weeks. The trial assessed the effectiveness of the drug in preventing brain injury in newborn babies.

The interventional (parallel assignment) clinical trial (NCT01913340) in a phase I and II demonstrates whether drug treatment given with hypothermia is a safe therapy and may in the future reduce the risk of neurologic deficits after HIE. The participants were divided into two groups; the experimental group received EPO α (Procrit) 1000 U/kg per dose given in five doses. The placebo group received 0,9% normal saline solution given in five doses [40,41,42]. The purpose of this clinical trial was to compare the severity of acute brain injury in neonates affected by HIE and treated with EPO vs. placebo by means of conventional MRI supported by diffusion-weighted imaging (DWI) and evaluating the correlation between volume of acute brain injury and outcome at 12 months of age. The data showed that the volume of injury was smaller in the erythropoietin-treated neonates with acute brain injury. Outcomes indicated low rate of deaths, no adverse events, and cases of moderate or severe neurodevelopmental impairments.

The r-hu-EPO were also used in the interventional (parallel assignment), non-randomized trial in a phase I and II (NCT00945789). The study was designed to assess the safety of the therapy in newborns due to the lack of literature data on the r-hu-EPO use in newborns with HIE. The participants (*n* = 45) received 2500 IU/kg per dose daily for 5 days. Patients were monitored with application of EEG and brain MRI. Additionally, the nitric oxide measurements in the blood were performed during enrollment into trial [43]. The data collected during the follow-up examinations indicated that at 6 months of age, infants in the EPO-treated group had fewer neurologic and developmental abnormalities; moreover, no seizures occurred.

In another trial, the drug darbepoetin α was tested in the interventional (parallel assignment), randomized study in a phase I and II (NCT01471015). The study assessed the safety and pharmacokinetics of early administration of darbepoetin alongside with hypothermia in newborn infants with moderate to severe birth asphyxia. The major goals of this research were to reduce mortality and lower the risk of long-term disabilities in infants with HIE who survive past the newborn period. The patients in the experimental group were divided into groups treated with either high-dose or a low-dose of darbepoetin. In the control group, participants received normal saline solution by the same way of delivery. The high-dose group obtained 10 mcg/kg/dose intravenously firstly within 12 h after delivery, and the second dose was given at 7 days old intravenously or subcutaneously. The low-dose group obtained 2 mcg/kg/dose intravenously firstly within 12 h after birth, and the second dose was given at 7 days old also intravenously or subcutaneously. The research was performed with simultaneous use of hypothermia. Pharmacokinetic data showed that the half-life (t1/2) of the first dose of darbepoetin (2 and 10 µg/kg) was 24 and 32 h, respectively. After seven days, the half-life was noted at 26 and 35 h, respectively. As a result, no statistically significant differences in mortality or adverse events were observed between study groups [44].

The next clinical trial involved the use of EPO β after HI injury in newborn babies. The trial (NCT01732146, NEUREPO)—interventional (parallel allocation), randomized in a phase III trial, attempted to assess the efficacy of high-dose EPO on outcomes at two years in term newborns with asphyxia undergoing cooling therapy. The neonates (120 participants of the study) received intravenous injections of 1000–1500 U/kg/dose, given in three doses every 24 h starting from within 12 h after birth. Half of the neonates received a placebo in normal saline. TH was also used in the above procedure. The results of the follow-up are still awaited.

The combination of EPO α (Epogen) and TH was used in the research (NCT02811263)—the interventional (parallel assignment), randomized in a phase III trial. The infants in the experimental group received a dose of 1000 U/kg of EPO α (Epogen) intravenously before 26 h of age and subsequently on days 2, 3, 4, and 7 of life. The placebo group was treated with normal saline solution (equal volume). The TH was performed for 72 h, started within 6 h after birth. The results indicate that administering multiple high doses of EPO with simultaneous TH to term and near-term newborns with moderate or severe HIE did not significantly alter the rates of death or neurodevelopmental impairment at 2 to 3 years of age. In addition, infants who received EPO were more likely to experience a greater number of serious adverse events than those who received placebo. The lack of benefit in this large trial casts doubt on the practice of administering multiple high doses of EPO to infants undergoing TH for HIE. In conclusion, in this trial, the administration of multiple high doses of EPO during the first week of life to neonates undergoing TH for moderate or severe HIE did not reduce the risk of death or neurodevelopmental impairment compared with placebo; a higher number of serious adverse events was also noted. The relative risk at the described trial was established as 1.03 (95% confidence interval [CI]). A higher number of serious adverse events were also noted than in the placebo group (0.86 vs. 0.67; relative risk, 1.26; 95% CI) [45].

The drug EPO β was also used in the interventional (parallel assignment), randomized research in a phase II and III (NCT02002039). In the study it was planned to test whether EPO improves the neurological outcomes of newborns with perinatal hypoxia-ischemia. The infants received the drug at 500 U/kg per dose every day for five doses as the treatment group. The placebo group received an equivalent dose of normal saline. The results of the study are not yet published.

In the recruiting study (NCT05395195), the investigators administered EPO to newborns with HIE in low and middle-income countries. This is an interventional (parallel assignment), randomized in a phase III clinical trial, in which newborns will obtain intravenously or subcutaneously a total of nine doses of EPO (500 U/kg per dose).

The first dose will be given within 6 h of birth, the second between 12 h to 24 h from the first dose. The further seven doses will be administered every 24 h from the second dose. The placebo group will receive only a standard neonatal intensive care.

The clinical trial (NCT03163589) is interventional (parallel assignment) and randomized in a phase III with unknown status involve testing EPO efficiency as a drug for infants injured by HIE. The study will estimate the neurodevelopment of neonates after few months of treatment. Half of the participants obtained intravenously 1000 U/kg of EPO in nine doses (on day 1, 2, 3, 5, 7, and 9) within 4–6 h after birth. Another half of patients enrolled into this trial receive the normal saline solution as placebo.

Overall, the results of the performed studies (if published) point to the contradictory effects, from beneficial even in relatively low doses (leading to limiting brain injury and improvement of neurodevelopmental process) to no significant effects in spite of administrating a high doses (even 2500 IU/kg) to HIE-newborns (Table 1). These reported inconclusive data contribute to the fact that the tested drug has not yet been introduced as a therapeutic strategy for HIE.

## 4. Melatonin

Melatonin (5-methoxy-N-acetyl-tryptamine), synthesized from tryptophan, is a naturally occurring substance produced in the pineal gland [46]. Its production is regulated by the hypothalamic suprachiasmatic nucleus, resulting in elevated levels at night and reduced levels during the day [47]. In mammals, endogenous melatonin primarily targets two G-protein coupled receptors, MT1 and MT2, which have high affinities for the hormone at concentrations of 1 nM and lower. These receptors are activated by the increased nighttime levels of circulating melatonin and are thought to mediate most of its physiological effects [48]. A crucial aspect of melatonin’s therapeutic potential for HIE is its significant antioxidant and anti-inflammatory properties, along with its ability to easily cross the BBB [49,50]. In the clinic, hypothermia combined with melatonin has been shown to be more effective in reducing serum oxidant levels in asphyxiated newborns than hypothermia alone (*p* < 0.001) [51]. The major clinical trials based on melatonin administration are listed in Table 2.

In the interventional (parallel assignment), randomized clinical trial in a phase I and II (NCT02071160), the researchers aimed to investigate the impact of adding melatonin-based treatment to whole-body cooling on brain injury and outcomes in neonates after perinatal asphyxia. The preparation of the drug to administration consisted of crushing the tablets of melatonin (1–3 mg/tablet; Puritan’s Pride, Oakdale, NY, USA) and dissolving subsequently in 5–10 mL of distilled water. The melatonin was administered to the experimental group, which also received whole body hypothermia for 72 h. The infants received five doses of 10 mg/kg melatonin using an orogastric tube. The control group was subjected to whole-body hypothermia for 72 h. The neurologic evaluations were performed at 6 months of life [51]. According to the published data, the participants of the melatonin/hypothermia group had fewer seizures on follow-up EEG. The brain examination with MRI scanning revealed less abnormalities in brain white matter. At 6 months, the melatonin treated neonates had improved survival without neurological or developmental abnormalities (*p* < 0.001).

The next trial (NCT02621944) is a dose escalation study. This interventional single-group assignment non-randomized in an early phase I is recruiting infants with HIE to evaluate the neuroprotective effects and optimal dosage of melatonin for infants undergoing TH. Participants will be divided into three groups. The first group will receive a 0.5 mg/kg enteral dose of melatonin, and the first dose will be administered via an enteral route within 12 h of life with a target of 6 h of life. Melatonin will be administered as a single dose to the first five participants, enabling investigators to assess if dosing frequency can be safe during hypothermia. If no safety concerns arise, the subsequent five subjects will receive multiple doses. Additionally, the patients will be monitored by MRI and for drug pharmacokinetics.

The second group will receive 3 mg/kg of melatonin, the third 5 mg/kg at the same schedule of administration as the first group. The long-term safety and neurodevelopment of infants will be estimated during 18–22 months of age.

In the withdrawn study (NCT01904786)—interventional (parallel assignment), randomized in a phase I—the drug melatonin (PureBulk, Roseburg, OR, USA) was administered to patients after HI. In the experimental group, 40 mg/kg of melatonin was administered orally (by nasogastric tube) every 8 h, for a total of six doses over a 48-h period. The control group received a placebo consisted of solvent solution without melatonin. Simultaneously with the drug administration, the TH was performed.

The next study (NCT03806816) is interventional (parallel assignment) and randomized, and the investigators used the melatonin (Buona Circadiem, Steve Jones, Sesto Fiorentino, Italy) to evaluate its neuroprotective properties in combination with whole-body cooling. The drug was administered 10 mg/kg daily in five doses with the current use of TH. The control group received only TH.

The mentioned list of evidence pointed to melatonin as a possible cure for consequences of neonatal HI (Table 3).

## 5. Allopurinol

Allopurinol is a xanthine oxidase inhibitor, which decreases the release of oxygen radicals, mostly of superoxide. Allopurinol is used for the treatment of hyperuricemia and gout or uric acid kidney stones. It is metabolized into oxypurinol by either xanthine oxidase (XO) or aldehyde oxidase (AO). Oxypurinol is excreted by the kidneys. Both allopurinol and oxypurinol act as competitive inhibitors of XO, blocking the conversion of hypoxanthine to xanthine and xanthine to uric acid; then, they lower blood uric acid levels [60]. Superoxide radicals cause damage to the mitochondria, leading to energy failure and apoptosis in neurons and glial cells after the reperfusion of hypoxic brain tissue, a condition known as reperfusion injury. This reperfusion results in further brain damage occurring within hours after birth and can impact significantly larger areas of brain tissue, compared to those initially affected during the event [61,62]. Superoxide production, which is limited by allopurinol, peaks within 30 min after birth, making early administration crucial for reducing reperfusion injury [63].

The multicenter trial (NCT03162653, ALBINO) is recruiting patients to assess the efficacy and safety of administering allopurinol immediately after birth to near-term infants with HIE, alongside hypothermic treatment. This interventional (parallel assignment), randomized study in a phase III is trying to determine whether newborns with asphyxia and early signs of HIE will benefit from early administration of allopurinol compared to a placebo, both in addition to standard care, with respect to long-term outcomes such as severe neurodevelopmental impairment or death within two years. The infants receive two doses of allopurinol—the first dose (20 mg/kg in 2 mL/kg sterile water) intravenously no later than 30 min postnatally. Second dose (10 mg/kg in 1 mL/kg sterile water) will be given after 12 h only if infants receive TH. If not, only the first dose will be administered. The infusion of the drug lasts 10 min. The control group will obtain mannitol as a placebo at the same volume, time intervals, and schedule as in intervention group [52,53]. The first patient was enrolled into the study on March 2018, while the current status of recruitment corresponds to 460 patients recruited. From all the newborns treated according to the study protocol, 300 recruited patients have reached 2 years postnatal age [54]. The pharmacokinetics of allopurinol and oxypurinol, as well of their pharmacodynamics and effects on inhibiting conversions of hypoxanthine and xanthine to uric acid was evaluated. The clearance (CL) of allopurinol was 0.83 L/h, and the volume of distribution (Vd) was 2.43 L. The CL and Vd of oxypurinol relative to the formation fraction (fm) were 0.26 L/h and 11 L, respectively. No difference in the CL of allopurinol and oxypurinol was found in patients treated with TH [55]. The investigators concluded that the dosing regimen used in the ALBINO trial is effective in terms of xanthine oxidase inhibition in the TH-treated neonates, as well as in the non-cooled group.

The terminated study (NCT00189007, ALLO-trial)—interventional (parallel assignment), randomized in a phase III hypothesis testing trial determined whether initiating of allopurinol treatment in the fetus experiencing (imminent) hypoxia through the mother during labor will be more effective in reducing free radical-induced brain damage following asphyxia. Fetal hypoxia was identified by an abnormal or non-reassuring fetal heart rate trace, significant ST-wave abnormalities, or abnormal fetal blood scalp sampling. Women received a single dose of 500 mg/kg allopurinol intravenously or a placebo immediately before delivery. After delivery no child died as a result of the study. Of all the cord blood samples analyzed, 95% (52 out of 55) had the target allopurinol plasma concentration at the time of delivery. The outcomes (138 out of the original 222 mildly asphyxiated children) showed that administering allopurinol to women in labor with suspected fetal hypoxia does not improve long-term developmental and behavioral outcomes at 5 years of age [56,57,58,59].

## 6. Sildenafil

Sildenafil is a phosphodiesterase type five (PDE5) inhibitor that reduces the breakdown of cyclic guanosine monophosphate (cGMP) into GMP. By preserving cGMP levels, sildenafil promotes smooth muscle relaxation and enhances blood flow to organs through the cGMP-dependent protein kinase activation of potassium channels [64]. Sildenafil induces vasodilation by acting on vascular smooth muscle and is commonly used to treat persistent pulmonary hypertension in newborns [65]. This drug is recognized for its neuroprotective effects, addressing secondary injuries and aiding in subsequent repair [66,67]. In the developing brain, sildenafil has been shown to enhance hemodynamic redistribution and increase vascular density, thereby improving microcirculation recruitment [68].

It also reduced brain tissue loss in rodent models of focal cerebral ischemia both early in life and in adulthood [69,70,71]. However, the neurobiological mechanisms behind these benefits remain unclear and are likely to involve glial cells [72].

The above-mentioned list of evidence pointed to sildenafil as a possible cure for consequences of neonatal HI (Table 4).

The study (NCT02812433, SANE-01)—interventional (parallel assignment), randomized in a phase I trial used a sildenafil to prevent outcomes of HIE. The investigators hypothesize that sildenafil may enhance neurodevelopmental outcomes in term asphyxiated newborns where hypothermia treatment has not successfully prevented brain injury. In the experimental group, the sildenafil was administered orally (2 mg/kg) twice a day from day 2 of life to day 9. The placebo group received the suspending vehicle Ora-blend at the same schedule as an interventional group [73]. According to results published in 2024, the combined rate of death or survival with severe neurodevelopmental impairment at 18 months was 57% in the sildenafil group and 100% in the placebo group.

The interventional (single group assignment), randomized study in a phase I (NCT05275725) aims to assess the feasibility, safety, and tolerability of administering sildenafil as a neuroprotective and neurorestorative strategy to enhance early brain development in a cohort of children with neonatal HIE in Uganda. The administration of sildenafil was divided into five groups: the first group obtained 4 mg/kg per day, the second 5 mg/kg, the third 6 mg/kg, the fourth 6 mg/kg, and the fifth 6 mg/kg during 12 h. The results of this trial are still awaited.

The study with the assigned number (NCT06098833) is recruiting infants who suffers from asphyxia. This interventional (parallel assignment), randomized research in phase II aims to determine if sildenafil is an effective treatment for repairing brain damage in newborns who experienced asphyxia at birth. It may also offer new solutions to enhance the future quality of life for these infants. The three groups of infants receive three different doses of sildenafil orally twice a day. The first group receives 2 mg/kg, the second 2.5 mg/kg, and the third 3 mg/kg for 7 days, starting on the second day to the ninth day. The control group obtain placebo as an Ora-Blend vehicle. The estimated completion date is October 2027, and the number of participants has been set at 60 newborns.

In the trial (NCT04169191)—interventional (single group assignment), in phase I—the investigators will identify the maximum tolerable dose of sildenafil and establish its pharmacokinetic and pharmacodynamic profile in asphyxiated neonates treated with hypothermia. The trial involves a dose escalation study (3 + 3) to the maximum daily dose of sildenafil (6 mg/kg/dose). Cohort 1 (3–6 neonates) receives the first dose of 2 mg/kg/dose, a second dose of 2.5 mg/kg/dose, and subsequent doses of 2.5 mg/kg/dose every 12 h (5 mg/kg/day). Cohort 2 (3–6 neonates) receives the first dose of 2 mg/kg/dose, a second dose of 2.5 mg/kg/dose, and third dose of 3 mg/kg/dose and subsequent doses of 3 mg/kg/dose every 12 h (6 mg/kg/day). Neonates will simultaneously undergo hypothermia.

## 7. Metformin

Metformin, a common treatment for diabetes type 2, is currently under investigation for its potential in treating metabolic syndrome [74]. The main pharmacological effects, which include antioxidant and anti-inflammatory actions, are primarily driven by the activation of AMP-activated protein kinase (AMPK). This activation can help modulate oxidative stress, prevent mitochondrial damage, and promote angiogenesis [75,76]. The metformin may be a neuroprotective agent. Some studies in animal models demonstrated neuroprotective effects after cerebral ischemia. Moreover, metformin enhances neurogenesis, maintains the integrity of the BBB in experimental strokes and supports remyelination in neonatal white matter after injury [77,78,79].

The influence of metformin on newborns with HIE was assessed in the terminated study (NCT05590676). This was an interventional (parallel assignment), non-randomized phase I trial, that tested the safety and feasibility of drug administered to newborns. As intended, five infants with HIE were included in the first dose cohort of 20 mg/kg metformin. Next, an additional five infants received the dose of 25 mg/kg metformin. Similarly, five preterm infants obtained the first dose cohort of 15 mg/kg metformin [80]. The study started in May 2023.

Descriptions of clinical trials from administration of melatonin to xenon have been compiled in a summary table (Table 5).

## 8. Glucocorticoids

Glucocorticoids (GCs) are a class of steroid hormones that include hydrocortisone. GCs are produced by the adrenal gland or can be administered externally as an anti-inflammatory or immunosuppressant treatment. The exogenous GCs are effective for reducing inflammation, but their use is linked to complications with long-term use. They can impact brain development through intracellular glucocorticoid and mineralocorticoid receptors. Preterm infants need support for basic bodily functions, such as mechanical ventilation for respiratory issues. The use of exogenous GCs in preterm infants in the neonatal intensive care unit (NICU) has been a highly debated and controversial topic in recent decades. GCs are often given to infants for reducing the incidence and severity of bronchopulmonary dysplasia, a significant risk factor for mortality and neurodevelopmental disabilities in preterm infants [92,93]. Several studies have demonstrated that administering GCs to the neonatal brain via intracerebroventricular injection or intranasal administration confer neuroprotection and mitigate brain damage following HI injury [94]. Based on the above evidence, GCs were introduced into clinical trials (Table 5).

In the completed study (NCT02700828)—an interventional (parallel assignment), randomized trial in phase II and III—the researchers proposed that the neonates who experienced asphyxia and underwent TH may develop relative adrenal insufficiency, leading to hypotension. The investigators planned to measure initial serum cortisol levels and examined the cardiovascular impact of administering low-dose hydrocortisone. The 32 participants were enrolled in a mentioned trial, and half of them received treatment with hydrocortisone (Solu-Cortef, Pfizer, Kalamazoo, MI, USA) intravenously in four doses of 0.5 mg/kg for 24 h in every 6 h during TH (max. for 72 h). The placebo group received isotonic sodium chloride solution in the same time intervals during TH. Before randomization, serum cortisol concentrations were low in both groups (median: 3.5 and 3.3 µg/dL; *p* = 0.87). Results indicated that administering hydrocortisone successfully increased blood pressure and reduced the need for inotropes in asphyxiated neonates with volume-resistant hypotension undergoing hypothermia treatment [81].

The recruiting study (NCT05836610)—an interventional (sequential assignment), non-randomized in phase IV—is recruiting newborns with neonatal HI for hydrocortisone treatment in systemic low blood pressure during TH. The investigators plan to measure initial baseline serum cortisol levels and monitor serial serum cortisol levels after administering hydrocortisone in cooled neonates. Participants, who will be enrolled to the standard treatment group, will receive intravenously 0.5 mg/kg of hydrocortisone (Solu-Cortef) in every 6 h. The adjusted hydrocortisone therapy will be determined according to the pharmacokinetic findings.

## 9. Topiramate

Topiramate (TPM) is an anticonvulsant drug used both in children and adults. Topiramate has an effective absorption, high bioavailability, and good tolerability. The mechanism of action focuses on many processes, including the inhibition of glutamate receptors. It is also considered as a neuroprotective drug [95,96]. The long-term effects of TPM on cognitive functions when administered early in life are still being studied. The short-term safety profile is sufficiently reassuring to warrant its inclusion in clinical trials investigating neuroprotective benefits [97]. The difference between effective and neurotoxic doses is larger using TPM than other antiepileptic drugs [98]. The short-term use appears to have minimal neurotoxic effects. Studies on asphyxiated animal models treated with TPM have shown no cognitive deficits, and similar results have been observed in epileptic neonatal rodents [99]. These beneficial effects are highly desirable to be obtained in the neonates who experienced HIE and, therefore, TPM has been involved in the designed clinical trials (Table 5).

The completed study (NCT01241019, NeoNati) was designed to confirm the safety of TPM administration and assessed whether combining TPM with hypothermia enhances the neuroprotective effects for treating neonatal HIE. In an interventional (parallel assignment), randomized study in phase II, 21 neonates (ten with moderate and eleven with severe HIE) received TPM (10 mg/kg) once a day via orogastric tube when the whole body cooling began. Newborns received one dose per day for the following 3 days after birth. The control group (12 infants with moderate and 11 severe HIE) was treated with whole-body hypothermia only for 72 h. However, a decrease in the occurrence of epilepsy was noted in newborns, who received both TPM and hypothermia treatment. The findings of this trial suggested that the given treatment is safe, but it does not lower the rate of death or severe neurological disabilities [82,83]. Investigators reported no statistically or clinically significant differences between both groups, and noted a reduction in the prevalence of epilepsy.

The goal of the following research was to convince whether TPM (as an anti-epileptic agent) improves the outcome of infants with neonatal HIE who are receiving whole body cooling. In this terminated study (NCT01765218)—interventional, randomized in phase I and II—participants received 5 mg/kg TPM in five doses as soon as possible. A total of 44 asphyxiated newborns were enrolled in the study. The patients enrolled into study (ten with moderate and eleven with severe HIE) were treated routinely with TH with additional administration of TPM. The placebo group underwent TH only (12 moderate and 11 severe HIE cases). The results indicate that TPM treatment was associated with a higher percentage of adverse events, and indicated a low rate of seizures and no mortality events.

## 10. Magnesium Sulphate

Magnesium sulphate is an N-methyl-D-aspartate receptor antagonist and is recognized for ability to reduce pain and decrease the need for anesthetics and analgesics. The mechanism of magnesium sulfate remains unclear [100,101,102]. A widely accepted theory claims that magnesium prevents excitotoxic damage by blocking NMDA receptors. These postsynaptic receptors are typically involved in strengthening synaptic connections through repeated activation, a process known as long-term potentiation, which is essential for memory function [103]. Magnesium is involved in vasodilation, hemostasis, and the maintenance of the BBB. It may also act as a neuroprotective agent in cases of acute stroke and brain hemorrhage [104,105]. Magnesium sulphate has been examined for its utility during HIE. Magnesium is frequently used as a supportive medication for newborns with HI injury, commonly alongside TH [106] (Table 5).

In the clinical trial (NCT04705142)—interventional (single group assignment) in phase II—the researchers administered magnesium sulphate to neonates with birth asphyxia. The participants received intravenously 250 mg/kg of magnesium sulphate in three doses—the first within 6 h after birth, and further doses after 24 h and 48 h. Infusion lasted 30 min. Infants in the control group received a placebo. The main objective of the study was to evaluate the beneficial effects of magnesium sulphate in neonates during birth asphyxia. Altogether, 41 infants were allocated to the group where they received intravenous magnesium sulphate infusion along with the routine medical care. As reported by investigators, they observed low rate of seizures and neurological problems, as well as a better ability to suck feed [84]. However, the study still lacks the long-term follow-up to confirm the effectiveness of treatment.

The drug magnesium sulphate is also planned to be used in a future recruiting study (NCT06342362). The study will investigate the neuroprotective effects of intravenous magnesium sulphate in neonates with HIE. In the interventional (parallel assignment), randomized study in phase IV—researchers will administer intravenously magnesium sulphate, or a placebo as a control.

In the interventional (parallel assignment), randomized study (NCT05707962) the investigators will try to assess the safety of magnesium sulphate given to newborn with HIE in low-income countries. The study is based off the application of 250 mg/kg substance intravenously in three doses for 30 min plus standard treatment (oxygen and fluid therapy along with anticonvulsants, if required) given 24 h apart. The placebo group receives standard treatment (oxygen and fluid therapy along with anticonvulsants, if required) plus 3.0 mL/kg of 10% dextrose water in three doses for 30 min. The neurodevelopmental outcome will be measured after 18 months of age.

## 11. Sovateltide

Sovateltide (IRL-1620) is a synthetic analog of endothelin 1 (ET-1). While ET-1 activates both endothelin A (ETARs) and endothelin B (ETBRs) receptors, sovateltide is a selective agonist for ETBRs. The endothelin family includes endothelins (ETs) and their receptors, which are powerful vasoconstrictor peptides that play crucial roles in managing the systemic and peripheral vascular systems [107]. Sovateltide administration significantly increased the expression of ETB receptors. This upregulation likely contributes to sovateltide’s neuroprotective effects. ETB receptors are essential for nervous system development. Additionally, sovateltide treatment boosted the expression of VEGF and NGF, which are critical for neuronal survival, neuroprotection, and regenerative processes such as growth, differentiation, or axonal outgrowth. This is a potential drug for enhancing neuronal recovery after HIE [108]. Sovateltide may help manage the pathophysiological progression of HIE by mitigating primary and secondary energy failure. It may be mediated via boosting hypoxia-induced survival factors and decreasing oxidative stress and cell death in the neonatal HI brain [109]. This drug is a “first in class” therapeutic for HIE (Table 5).

In the recruiting study (NCT05514340)—interventional (parallel assignment), randomized in phase II—the investigators plan to conduct a clinical study to evaluate the safety and efficacy of IRL-1620 therapy in combination with supportive management in neonates with perinatal asphyxia. TH is currently the only treatment for HIE and has limited success. Studies suggest that sovateltide may offer additional benefits for these patients. The neonates receive intravenously 0.3 µg/kg of IRL-1620 during 1 min bolus every 3 h on day 1, day 3, and day 6 post randomizations. The control group receives normal saline solution in the same schedule.

## 12. Cerebrolysin

Cerebrolysin is a compound of amino acids and peptides that mimic the effects of neurotrophic factors. This pharmacological substance has demonstrated beneficial outcomes administered after ischemic stroke, while maintaining a safety profile. It helps reduce the levels of procoagulant, prothrombotic, and proinflammatory mediators also preserving the function and health of the cerebral microvasculature post-ischemia. BBB injuries caused by fibrin molecules and thrombolysis lead to the production of numerous inflammatory cytokines. This drug can enhance the therapeutic efficacy and safety of thrombolytic agents, as well as thrombectomy by protecting the BBB [110]. Cerebrolysin is an agent with a complex mechanism of action, known for its proven multimodal and pleiotropic effects. It offers immediate neuroprotection and promotes long-term neuroregeneration by activating endogenous responses. These effects have been observed in various cerebrovascular and neurodegenerative diseases [111] (Table 5).

The completed study (NCT01059461)—interventional (single group assignment) in phase II—aimed at pharmacological therapy enhanced psychomotor outcomes in infants with moderate to severe HIE following hospital discharge. The IRL-1620 was administered intramuscularly 0.1 mL/kg twice a week, and each infant (aged 3–6 months) received 10 injections after discharge from the NICU. Neurodevelopmental assessments were conducted prior to therapy, then at 3 and 6 months post-therapy. Outcomes pointed to improved social and speech composite, minimal side effects after treatment, and no changes in seizures frequency and duration. After three months, improvements in social (*p* < 0.01) and speech (*p* = 0.02) scores were observed in the placebo group. In the treated group, there was an enhancement in social (*p* < 0.001), speech (*p* < 0.001), and total (*p* < 0.001) scores [85].

## 13. N-Acetylcysteine and Calcitriol

N-acetylcysteine (NAC) is a synthetic derivative of the endogenous amino acid L-cysteine and a precursor of glutathione (GSH). NAC modulates oxidative stress and various other pathophysiological processes involved in disease. These processes include mitochondrial dysfunction, apoptosis, inflammation, as well as indirect effects on neurotransmitters like glutamate and dopamine [112,113]. NAC is an antioxidant and free radical scavenger that boosts intracellular levels of GSH, a crucial component in the cellular defense mechanisms against oxidative stress [114]. Enhancing intracellular antioxidant reserves in the brain immediately after HI may be crucial for stopping the progression of neural cell death and increasing neuroprotection in infants with HIE, who do not benefit from hypothermia treatment [115]. Active calcitriol (vitamin D) is a neurosteroid that plays a role in neuroplasticity, myelination, and also in normal brain development [116,117,118] (Table 5).

The combination of NAC and vitamin D therapy was performed in the clinical trial (NCT04643821). In this interventional (single group assignment)—non-randomized in early phase I—the investigators used a dose escalating scheme. The research aimed to evaluate the distribution and safety of NAC combined with active vitamin D in neonates presenting within 6 h of a HI event, who have undergone moderate hypothermia for 72 h as per routine protocol. The first group received intravenously 25 mg/kg of NAC (Acetadote, Cumberland Pharmaceuticals, Nashville, TN, USA) and vitamin D (Calcitriol Injection USP. Akron Inc., Lake Forest, IL, USA) 0.05 mcg/kg intravenously every 12 h for 10 days. The second group also received NAC in the same dosage, but vitamin D in regiment 0.03 mcg/kg every 24 h. The third group was given a higher dose of NAC: 40 mg/kg every 12 h and vitamin D 0.03 mcg/kg every 24 h. The treatment started within 6 h of birth. Development was followed for >24 months [86,87]. The neuroimaging performed on 24 infants on 5–6 days of life by means of magnetic resonance spectroscopy, indicates that NAC rapidly replenishes GSH, which level is significantly lowered after HI due to persistent oxidative stress. Thus, the combined administration of NAC and vitamin D may act synergistically to mitigate oxidative stress and contribute to neuroprotection of the injured neonatal brain. Results showed that NAC increased GSH levels in brains after HIE.

## 14. Vitamins and Ibuprofen

Ascorbic acid (vitamin C) is an antioxidant, combating oxidative stress through electron transfer or donation. It has several active forms, and L-ascorbic acid is the most extensively studied and biologically active [119]. Tocopherol (vitamin E) is a lipophilic antioxidant and anti-inflammatory agent with potential neuroprotective properties [120,121]. Neonatal neurological injuries can arise or worsen due to oxidative stress and inflammation [122]. Vitamin E deficiency can lead to nervous system development, as it is crucial for normal embryonic development, neurogenesis, and cognition [123]. However, the evidence supporting vitamin E’s neuroprotective efficacy remains controversial. Although numerous rodent studies have investigated effects of vitamin E application, their clinical translation has shown varying degrees of effectiveness [124,125]. Ibuprofen, a non-steroidal anti-inflammatory drug (NSAID), alleviates pain and reduces inflammation by inhibiting the cyclo-oxygenase enzyme (COX). There are two forms of this enzyme: COX-1—produces prostanoids and thromboxane A2 from arachidonic acid and COX-2, which is naturally found in certain tissues such as brain, kidneys, and female reproductive tract. COX-2’s role involves the production of prostaglandins, which are key mediators of processes associated with pain, inflammation, and fever reactions [126] (Table 5).

Accordingly, the trial (NCT01743742), interventional (single group assignment) in phase IV, was aimed at investigating the impact of high-doses vitamins C and E administered orally on the first day after birth in newborns with HIE. The study aimed the effect of designed treatment on reducing morbidity and modulation of neurodevelopmental outcomes. According to the study protocol, infants received orally (via infant feeding tube) 200 IU of vitamin E (Evion) and 250 mg of vitamin C (Limcee) at 6 h after birth. The vitamin E was administered once, while vitamin C twice, after a 24 h interval. The detailed results of the study are still to be published.

In the study (NCT00624871)—interventional (parallel assignment) and randomized the investigators established the efficacy of combination two drugs when combined to decrease brain injury. The vitamin C was administered intravenously at a dosage of 100 mg/kg for 3 days and the ibuprofen orally 10 mg/kg on first day, then 5 mg/kg on the second and third day of life. Infants in the control group received an equivalent amount of placebo [88]. As reported, there was no severity in HIE. The incidence of mortality was 33%, the incidence of neurological abnormalities at hospital discharge was 55%, and the incidence of developmental delay at six months of age was 40%.

## 15. Caffeine Citrate

Caffeine (methyl theobromine) is a commonly used natural stimulant belonging to the methylxanthine class. Caffeine works on adenosine receptors [127]. At non-toxic doses, caffeine mainly works by blocking adenosine receptors. In the brain, caffeine affects A1 and A2A adenosine receptors, with its typical concentrations influencing neuronal function solely through these receptors. Moreover, the neuroprotective effects of caffeine in the brain are entirely due to its antagonism of A2A adenosine receptors, as detailed in recent reviews [128,129,130,131]. In animal models of HIE, caffeine has been shown to mitigate white matter brain damage [132]. Even though a therapy with caffeine is used in NICU to treat the apnea of prematurity [133], to date there are no data about the efficacy and safety of caffeine therapy in newborns after HI injury (Table 5).

In the active study (NCT03913221)—interventional (sequential assignment), non-randomized in phase I—the caffeine citrate (Cafcit) is used as an adjuvant therapy to improve neurodevelopmental outcomes. Newborns undergoing TH as a treatment for HIE receive caffeine as a loading dose of 20 mg/kg, followed by two daily doses of 5 mg/kg (*n* = 9) or 10 mg/kg (*n* = 8). Apparent caffeine clearance was planned to be measured in samples collected after the first dose and up to 72 h after final dose. Methylxanthines, the drug class to which caffeine belongs, have demonstrated protective effects against acute kidney injury in the context of HIE, so there is a chance to improve health of infants after HI [89]. Results of the study reported some situations of adverse events after treatment.

In the next study (NCT06448780), the caffeine citrate will be also performed as a drug for HIE. In this interventional (sequential assignment), non-randomized study in phase I, the participants in first cohort will obtain 10 mg/kg of caffeine citrate (Cafcit) twice a day (loading dose 20 mg/kg). The second cohort will obtain also 10 mg/kg in the same schedule with the loading dose 30 mg/kg. The trial focuses on optimizing an appropriate drug dose with undergoing TH.

The withdrawn study (NCT05295784) investigated the blood levels of caffeine in neonates with HIE undergoing TH. The investigators expected caffeine to be a safe and effective therapy. In an interventional (sequential assignment), non-randomized trial in phase I, three different doses of caffeine citrate (Cafcit) were to be used. Participants enrolled in the study (a total of 18 neonates). Each neonate received a single dose of caffeine in the first 24 h of life: the first six neonates were to be treated with low dose (5 mg/kg), a medium dose (15 mg/kg) was administered to the next six newborns, and finally, the six patients were treated with the highest dose as like 20 mg/kg. Accordingly, however, to the update posted on May 2024, the study has been withdrawn.

## 16. 2-Iminobiotin

2-Iminobiotin (2-IB) is a vitamin H, which contains guanidine and free carboxyl groups. These groups enable 2-IB to bind within the active site of NOS, similar to L-arginine. Studies have demonstrated that 2-IB inhibits iNOS and nNOS activity in a concentration-dependent manner, leading to a decrease in NO production [134]. 2-IB provides neuroprotection by inhibiting the neuronal and inducible forms of NOS. The aim to use it in HIE treatment was to modulate the pathophysiological pathways activated by oxygen deprivation in the brain shortly after HI [135,136]. The first study of 2-IB in newborns indicated that both single and multiple doses of 2-IB (up to 6 doses of 6 mg/kg) were found to be safe and well-tolerated in healthy male subjects, whether administered with or without Captisol (solubilizing agent). 2-IB demonstrated high clearance, a volume of distribution slightly greater than total body water and linear pharmacokinetics, that were unaffected by the presence of Captisol [137] (Table 5).

The research registered as (NCT01626924) aimed to evaluate short term efficacy, pharmacokinetics and safety of 2-IB. In the interventional (single group assignment) study in a phase II the researchers hypothesized whether the drug may reduce brain damage following oxygen deprivation and if it proves to be safe. The health condition of children should be assessed within the first two years of life. 2-IB was administered on a start dosage of 0.2 mg/kg intravenously, and six doses within 20 h. The results of the study are waiting to be published.

## 17. Citicoline

Citicoline is a form of cytidine 5-diphosphocholine (CDP-choline), formed during the rate-limiting step of transforming choline into phosphatidylcholine. When citicoline is administered orally, it is quickly absorbed as it is broken down in the gut into cytidine and choline [138,139]. In case of HIE in newborns, citicoline aids in neurorepair by targeting various stages of the cascade, it protects damaged tissue from both immediate and delayed mechanisms of brain injury. It works by inhibiting glutamate accumulation, regenerating cell membranes and enhancing brain plasticity [140,141] (Table 5).

Citicoline as a drug was used in an interventional (single group assignment), randomized study in a phase III (NCT03181646). Infants in experimental group received 15 mg/kg intravenously of citicoline until oral feeds established. The control group obtained only supportive care. The estimated date of study completion was indicated as the end of 2017; however, to date, no results have been posted.

## 18. RLS-0071

RLS-0071 is named as Peptide Inhibitor of Complement C1 (PIC1). This novel peptide has anti-inflammatory activity and consists of 15 amino acids, and inhibits cellular and humoral inflammation. RLS-0071 blocks many cellular pathways like complement activation, inhibits myeloperoxidase (MPO) activity, and neutrophil extracellular traps (NETs) formation. This peptide was establish as the therapy for HIE and neutrophilic pulmonary diseases [142,143] (Table 5).

The safety and tolerability of RLS-0071 will be evaluated in the treatment of newborns with HIE, registered as study number (NCT05778188). In an interventional (parallel assignment), randomized in a phase II study, the first cohort (moderate and severe HIE) of newborns will receive 3 mg/kg of RLS-0071 every 8 h. The second cohort (moderate and severe HIE) will be administered 10 mg/kg every 8 h and the third cohort (moderate and severe HIE) will obtain 20 mg/kg also every 8 h. The placebo cohort will receive 0.9% sodium chloride at the same scheme as like RLS-0071 group. The study is based on infusion of ten doses over 72 h. Infants will be provided with standard care, including TH. The long-term observation will last until 24 months of children age.

## 19. Xenon

Xenon is an odorless medical gas used frequently in adults during anesthetic inhalation. In neonates with HIE, xenon has a quick onset of action when inhaled and provides anticonvulsant effects [144]. In animal models, xenon significantly decreased brain injury and indicated an additive neuroprotective effect when performed with hypothermia immediately after the insult [145,146,147]. On a molecular level, this gas is an antagonist of NMDA receptors and prevents glutamate-mediated excitotoxicity. It interacts with phenylalanine and binds to the glycine receptor [148,149,150]. Xenon provides most neuroprotective benefits by inhibiting NMDA receptors and reducing apoptotic cell death during phases of reperfusion injury (early and late) [136]. Xenon was also shown to enhance the production of hypoxia-inducible factor 1α (HIF-1α) [151] (Table 5).

In the completed trial (NCT00934700), newborns with HIE symptoms were classified for administration of xenon during TH. The interventional (parallel assignment), randomized study tried to assess whether inhaled gas with simultaneous hypothermia treatment exerts any neuroprotective effects on cerebral structure and metabolism. Neonates enrolled in the experimental group received endotracheal inhalation of 30% xenon gas (balanced with oxygen and air) for 24 h. At the same time TH was applied for 72 h and started 6 h after birth. In the placebo group, newborns obtained TH and standard intensive care. As a result, study has shown low rate of adverse events, and few deaths occurred [90].

This study (NCT02071394) investigated the impact of inhaled xenon gas on treating newborn infants with HIE when used alongside the conventional cooling therapy. The hypothesis suggested that the combination of xenon (LENOXe) and cooling offers better neuroprotection compared to cooling therapy alone. In the interventional (parallel assignment), randomized in a phase II study, newborns in the experimental group (five newborns) inhaled 50% of xenon gas for 18 h staring within 5 h after birth plus 72 h of whole body cooling. The placebo group obtained only standard hypothermia within 3 h after birth for 72 h [91]. Authors concluded that xenon delivery during ambulance retrieval was feasible, enabling immediate start of treatment.

To sum up, the conclusions from to-date clinical trials indicate that cooling plus xenon was not associated with reduced mortality at the latest follow-up (eighteen months to three years of age). Additionally, investigators noted no substantial differences between groups in terms of the analyses of the selected biomarkers of brain damage and occurrence of seizures during primary hospitalization [144].

## 20. Discussion

Neonatal HIE leads to several neurological impairments, its consequences resulting in psychomotor disabilities, and in some cases the death of a child. Due to the extent of the injury, its severity, and the brain structures affected by the injury, adequate neonatal medical care is a challenge in countering the effects of the HIE. Randomized clinical trials (RCTs) are very influential in clinical practice, they provide high-quality scientific evidence (Table 6). RCTs significantly shape medical guidelines and treatment decisions. However, pediatric research faces some problems, such as ethical issues, difficulties in recruiting children for research, and problems with research funding. Adequate staffing of researchers with the knowledge and competence to conduct such studies also becomes a challenge. Alternatives include cohort studies, which are conducted when RCTs are not feasible [152]. One of the primary challenges lies in translating promising results from animal studies to human applications. Due to biological differences between species, some positive preclinical outcomes may not hold true in human neonates, emphasizing the need for more rigorous studies, whether in animals or humans [153]. Because of the multimodal and complex nature of HIE, therapies should target different pathways to ensure treatment effectiveness. Many drugs are currently being assessed as adjuncts to TH, which is now routinely applied in the clinical entities as a first aid to limit spreading of the brain injury.

So far, none of the drugs used has been shown in clinical trials to be fully effective in the long term. There is great hope, that drugs such as melatonin, allopurinol, citicoline, RLS-0071, metformin, and caffeine citrate will be used in clinical settings in the near future. Sovateltide, which showed tolerability, efficacy, and safety in a clinical trial for ischaemic stroke in adults [154], also reduced neuronal brain damage during HIE in a rat model in preclinical studies [108]. Sovateltide has been developed as a first-in-class drug that has also shown the ability to exert multimodal effects, which are critical for controlling HI injury and promoting recovery, by stimulating neuronal regeneration and restoring function in the brain affected by HIE. Drugs that exhibit multimodal functions may be an effective therapy to treat HIE injury.

The main obstacle to comparing the results of clinical trials based on pharmacological treatments is the lack of uniform protocols in terms of dose, time of administration after the HI insult, number of applications, and the choice of patients receiving therapy. Long-term follow-up (at least up to 2–5 years) is also lacking. Parameters such as brain development, behavioral, and cognitive function in the follow-ups are measured according to selected, non-uniform criteria. Therefore, it seems necessary to provide standardized criteria when registering a clinical trial. In addition, the results of completed trials are not often made public, either on the clinical trial website or in scientific publications. Moreover, the studies are often discontinued without officially stating the reason [155]. These shortcomings significantly delay and hinder the development of effective therapies, and, despite so many clinical trials, there are still no pharmacological therapies to limit neurodegenerative processes and promote neuroregeneration. In conclusion, there is a need to identify therapeutic approaches to reduce the neurological deficits associated with HIE. The work of researchers and clinicians is crucial to understanding the disease and improving survival and quality of life for neonates affected by HIE, and the availability of an off-the-shelf cellular therapy for HIE could improve outcomes for these children.

## Figures and Tables

**Table 1:** Clinical trials based on administration of erythropoietin to patients who experienced perinatal asphyxia.

NCT Number/ Country	Recruitment Status	Pharmacological Substance	Administration	Schedule of Administration	Apgar Score	Age	Number of Participants	Reported Outcomes	References
NCT00719407 United States	Completed (study completion—November 2012)	Erythropoietin α (Procrit)	Intravenously	Six doses (250, 500, 1000, 2500 U/kg per dose) first dose < 24 h, subsequent at 48-h intervals	≤5 at 10 min	Up to 24 h	24	No deaths reported, moderate to severe disabilities occurred, cases of brain injury on neonatal MRI	[38,39]
NCT03079167 Australia New Zealand Singapore	Completed (study completion—April 2024)	Erythropoietin α (Procrit, Epogen)	Intravenously	1000 IU/kg (capped up to 4000 IU/kg per day) on 1, 2, 3, 5 and 7 days after birth	≤5 at 10 min	Up to 23 h	313	Not reported	-
NCT00808704 China	Completed (study completion—July 2008)	Recombinant human erythropoietin (r-hu-EPO)	Subcutaneously and intravenously	Either 300 U/kg and 500 U/kg first subcutaneously then intravenously for every day for 2 weeks	≤5 at 5 min	1 h to 48 h	167	Not reported	-
NCT01913340 United States	Completed (study completion—September 2016)	Erythropoietin α (Procrit)	Intravenously	1000 U/kg per dose; five doses	≤5 at 10 min	30 min to 24 h	50	Low rate of deaths, not reported adverse events and moderate or severe neurodevelopmental impairments	[40,41,42]
NCT00945789 Egypt	Completed (study completion—June 2009)	Recombinant human erythropoietin	Subcutaneously	2500 IU/kg per dose daily for 5 days	≤3 at 5 min	Up to 24 h	45	No seizures reported, MRI did not differ between groups, lower neurologic and developmental abnormalities	[43]
NCT01471015 United States	Completed (study completion—January 2014)	Darbepoetin α	Intravenously or subcutaneously	High dose: 10 mcg/kg/dose first dose within 12 h of delivery, second dose given at 7 days old; low dose: 2 mcg/kg/dose first dose within 12 h of delivery, second dose given at 7 days old	≤5 at 10 min	Up to 12 h	30	Adverse events occurred	[44]
NCT01732146 France	Completed (study completion—February 2017)	Erythropoietin β	Intravenously	1000–1500 U/kg/dose given three times every 24 h, first dose within 12 h of delivery	≤5 at 10 min	Up to 12 h	120	Not reported	-
NCT02811263 United States	Completed (study completion—April 2022)	Erythropoietin α (Epogen)	Intravenously	1000 U/kg per dose before 26 h of age and at 2, 3, 4, and 7 days of age	≤5 at 10 min	Up to 24 h	500	Higher number of serious adverse events, no changes in deaths/neurodevelopmental impairments	[45]
NCT02002039 India	Completed (study completion—June 2016)	Erythropoietin β	Intravenously	500 U/kg per dose every day for five doses starting at first 6 h of life	≤5 at 10 min	Up to 6 h	100	Not reported	-
NCT05395195 Bangladesh India Sri Lanka UK-sponsor	Recruiting (study start—December 2022)	Erythropoietin	Intravenously or subcutaneously	500 U/kg per dose. First dose within 6 h of birth; second between 12–24 h from the first dose; subsequent seven doses every 24 h from the second dose; total nine doses	<6 at 5 min	1 h to 6 h	504	Not reported	-
NCT03163589 Egypt	Unknown (study completion—June 2022)	Erythropoietin	Intravenously	1000 U/kg within 4–6 h after birth on days 1, 2, 3, 5, 7, and 9 (nine doses)	<5 at 10 min	Up to 24 h	40	Not reported	-

Simultaneously with the administration of the drug, the whole-body TH is applied. Data coming from that study are still awaited.

**Table 2:** Clinical trials based on administration of melatonin as a potential cure for HIE in affected neonates.

NCT Number/ Country	Recruitment Status	Pharmacological Substance	Administration	Schedule of Administration	Apgar Score	Age	Number of Participants	Reported Outcomes	References
NCT02071160 Egypt	Completed (study completion—December 2013)	Melatonin (Puritan’s Pride)	Intraesophageally	10 mg/kg daily, five doses started at enrollment	≤3 at 5 min	Up to 6 h	45	Fewer seizures, less white matter abnormalities on MRI, at 6 months improved survival without neurological or developmental abnormalities	[51]
NCT02621944 United States	Recruiting (study start—November 2016)	Melatonin	Enterally	Dose escalation study—0.5 mg/kg; 3 mg/kg; 5 mg/kg enteral route within 12 h with a target of 6 h of life	<5 at 10 min	Up to 6 h	70	Not reported	-
NCT01904786 United States	Withdrawn (lack of enrollment for suitable candidates) (study completion—November 2018)	Melatonin (PureBulk)	Orally	40 mg/kg every 8 h for a total of six doses given over 48 h	No Apgar	1 h to 8 h	0	Not reported	-
NCT03806816 Italy	Unknown (study completion—December 2022)	Melatonin (Buona Circadiem)	Enterally	10 mg/kg/daily five doses	<5 at 10 min	1 h to 6 h	100	Not reported	-

**Table 3:** Clinical trials based on administration of allopurinol as a potential cure for HIE in affected neonates.

NCT Number/ Country	Recruitment Status	Pharmacological Substance	Administration	Schedule of Administration	Apgar Score	Age	Number of Participants	Reported Outcomes	References
NCT03162653 Austria Belgium Estonia Finland German Italy Netherlands Norway Poland-withdrawn Portugal-withdrawn Spain Switzerland	Recruiting (study start—March 2018)	Allopurinol (powder for injection)	Intravenously	First dose—20 mg/kg in 2 mL/kg sterile water no later than 30 min postnatally; second dose—10 mg/kg in 2 mL/kg sterile water 12 h thereafter	≤5 at 10 min	Up to 45 min	760	Not reported	[52,53,54,55]
NCT00189007 Netherlands	Terminated (study completion—December 2016)	Allopurinol (Acepurin)	Intravenously	One dose 500 mg/50 mL intravenously to women before delivery	Not reported	Child, adult, older adult	222	Not reported long-term developmental and behavioral outcomes at 5 years of age	[56,57,58,59]

**Table 4:** Clinical trials based on administration of sildenafil as a potential cure for HIE in affected neonates.

NCT Number/ Country	Recruitment Status	Pharmacological Substance	Administration	Schedule of Administration	Apgar Score	Age	Number of Participants	Reported Outcomes	References
NCT02812433 Canada	Completed (study completion—January 2022)	Sildenafil	Orally	2 mg/kg/dose twice a day for 7 days (from day 2 of life to day 9 of life)	Apgar ≤ 5 at 10 min	0 min to 48 h	28	Few severe neurodevelopmental impairments occurred	[69]
NCT05275725 Uganda	Recruiting (study start—July 2022)	Sildenafil	Not reported	Five groups: first group—4 mg/kg; second group—5 mg/kg; third group—6 mg/kg; fourth group—6 mg/kg; fifth group—6 mg/kg	Apgar ≤ 5 at 5 min	0 days to 18 months	30	Not reported	-
NCT06098833 Canada	Recruiting (study start—July 2024)	Sildenafil (Viagra)	Orally	Three doses twice a day: 2 mg/kg, 2.5 mg/kg and 3 mg/kg (day 2 to day 9)	Apgar ≤ 5 at 10 min	0 min to 48 h	60	Not reported	-
NCT04169191 Canada	Active, not recruiting (study start—September 2019)	Sildenafil citrate	Not reported	Dose escalation study (3 + 3) with maximum dose 6 mg/kg/dose every 12 h	Apgar ≤ 5 at 10 min	0 days to 2 days	20	Not reported	-

**Table 5:** Clinical trials based on treatment with the selected drugs as potential cures for HIE in affected neonates.

NCT Number/ Country	Recruitment Status	Pharmacological Substance	Administration	Schedule of Administration	Apgar Score	Age	Number of Participants	Reported Outcomes	References
Metformin									
NCT05590676 Canada	Terminated (low recruitment) (study completion—February 2024)	Metformin	Intravenously	4 group (15 mg/kg, 20 mg/kg, 25 mg/kg)	Not reported	Up to 3 months	1	Not reported	[80]
Glucocorticoids									
NCT02700828 Hungary	Completed (study completion—December 2022)	Hydrocortisone (Solu-Cortef)	Intravenously	4 × 0.5 mg/kg for 24 h (in every 6 h)	≤5 at 10 min	Up to 72 h	32	Effectiveness in raising blood pressure, decreasing inotrope requirement	[81]
NCT05836610 Hungary	Recruiting (study start—September 2021)	Hydrocortisone (Solu-Cortef)	Intravenously	0.5 mg/kg (in every 6 h)	Not reported	Up to 72 h	50	Not reported	-
Topiramate									
NCT01241019 Italy	Completed (study completion—December 2013)	Topiramate (Topamax, Janssen-Cilag, Cologno Monzese, Milan, Italy)	Orogastrically	10 mg/kg once a day, total of three doses per patient started on the third day of life	<5 at 10 min	36 weeks and older	64	No statistically or clinically significant differences between both groups, reduction in the prevalence of epilepsy	[82,83]
NCT01765218 United States	Terminated (study completion—January 2022)	Topiramate (Topimax, Topiragen)	Enterally	5 mg/kg in five doses as soon as possible	<5 at 10 min	Up to 6 h	34	Low rate of seizures, no mortality or adverse events reported	-
Magnesium sulphate									
NCT04705142 Pakistan	Completed (study completion—December 2020)	Magnesium sulphate	Intravenously	250 mg/kg, total of three doses, first within 6 h of life, second after 24 h, third after 48 h	Not reported	Up to 6 h	200	Low rate of seizures and neurological problems, better ability to suck feed	[84]
NCT06342362 Pakistan	Not yet recruiting (study start—April 2024)	Magnesium sulphate	Intravenously	Not reported	Not reported	1 day to 30 days	102	Not reported	-
NCT05707962 Pakistan	Not yet recruiting (study start—March 2023)	Magnesium sulphate	Intravenously	250 mg/kg at 3 doses given 24 h apart, not later than 24 h of life	Not reported	1 h to 24 h	178	Not reported	-
Sovateltide									
NCT05514340 India	Recruiting (study start—September 2023)	Sovateltide (IRL-1620)	Intravenously	0.3 µg/kg in 3 doses, every 3 h on day 1, day 3, and day 6 post randomization	<5 at 10 min	Child, adult, older adult	40	Not reported	-
Cerebrolysine									
NCT01059461 Egypt	Completed (study completion—September 2013)	Cerebrolysin	Intramuscularly	0.1 mL/kg twice weekly, ten injections	<5 at 10 min	3 months to 6 months	40	Improved social and speech composite, minimal side effects, no changes in seizures frequency or in duration	[85]
N-acetylcysteine and calcitriol									
NCT04643821 United States	Completed (study completion—March 2020)	N-acetylcystein (NAC), Vitamin D (calcitriol)	Intravenously	First group: NAC 25 mg/kg every 12 h, calcitriol 0.05 mcg/kg every 12 h for 10 days; second group: NAC 25 mg/kg every 12 h, calcitriol 0.03 mcg/kg every 24 h for 10 days; third group: NAC 40 mg/kg every 12 h, calcitriol 0.03 mcg/kg every 24 h for 10 days; starting within 6 h after birth	<5 at 5 min	Up to 6 h	30	NAC increased GSH levels in brains after HIE	[86,87]
Vitamins and ibuprofen									
NCT01743742 India	Completed (study completion—October 2013)	Vitamin E (Evion), vitamin C (Limcee)	Orally	200 IU single dose of vit E, 250 mg vit C (two doses at 24 h interval)	<6 at 5 min	1 min to 6 h	95	Not reported	-
NCT00624871 Egypt	Completed (study completion—April 2009)	Vitamin C, ibuprofen	Intravenously/orally	Vitamin C: 100 mg/kg/dose every day for 3 days, ibuprofen: 10 mg/kg on first day, 5 mg/kg on second and third day for 3 days	<6 at 5 min	Up to 2 h	60	No severity in HIE, no difference in the incidence of neurological abnormalities, no developmental delay	[88]
Caffeine citrate									
NCT03913221 United States	Active, not recruiting (study start—August 2019)	Caffeine citrate (Cafcit)	Intravenously	Low-dose: caffeine citrate 5 mg/kg twice a day; high-dose: 10 mg/kg twice a day within 24 h of delivery (loading dose 20 mg/kg)	Not reported	Up to 24 h	17	Reported adverse events	[89]
NCT06448780 United States	Not yet recruiting (study start—July 2024)	Caffeine citrate (Cafcit)	Intravenously	Low-dose: caffeine citrate 10 mg/kg twice a day (loading dose 20 mg/kg); high-dose: 10 mg/kg twice a day (loading dose 30 mg/kg) within 24 h of delivery	Not reported	Up to 24 h	16	Not reported	-
NCT05295784 United States	Withdrawn (data no longer support this study) (study completion—May 2024)	Caffeine citrate	Intravenously	Low dose: 5 mg/kg; medium dose: 15 mg/kg; high dose: 25 mg/kg once in the first 24 h of life	Not reported	0 h to 24 h	0	Not reported	-
2-iminobiotin									
NCT01626924 Turkey	Terminated (study completion—October 2014)	2-iminobiotin	Intravenously	0.2 mg/kg/dose/6 doses given in 20 h	≤5 at 10 min	Up to 6 h	6	Not reported	-
Citicoline									
NCT03181646 Pakistan	Unknown status (study completion—December 2017)	Citicoline	Intravenously	15 mg/kg/dose	Not reported	1 h to 14 days	50	Not reported	-
RLS-0071									
NCT05778188 United States	Recruiting (study start—July 2023)	RLS-0071	Intravenously	3 mg/kg, 10 mg/kg, 20 mg/kg every 8 h, total 10 doses for 72 h	≤5 at 10 min	Up to 10 h	42	Not reported	-
Xenon									
NCT00934700 United Kingdom	Completed (study completion—September 2014)	Xenon gas (LENOXe)	Endotracheally	30% xenon gas for 24 h	<5 at 10 min	1 h to 12 h	92	Low rate of adverse events, cases of deaths occurred	[90]
NCT02071394 United Kingdom	Completed (study completion—April 2020)	Xenon gas (LENOXe)	Endotracheally	50% xenon gas for 18 h within 5 h after birth	≤5 at 10 min	Child, adult, older adult	50	No reduction of mortality, no substantial differences between groups after analysis of brain damage biomarkers, no seizures occurred during primary hospitalization	[91]

**Table 6:** Advantages and pitfalls of studied pharmacological treatments.

Drug	Advantages	Pitfalls
EPO	Neuroprotective agent; enhances neuronal and glial migration around the injured site; increases neurodevelopment in children affected by HIE; prevents brain injury in newborns; low rate of deaths; fewer neurologic and developmental abnormalities; no significant side effects	Disabilities and brain injuries, ranging from moderate to severe, are sometimes experienced, and a high number of adverse events were reported.
Melatonin	Antioxidant and anti-inflammatory agent; ability to easily cross the BBB; fewer seizures; improves survival without neurological and/or developmental abnormalities; less white matter abnormalities	-
Allopurinol	Decreases the release of oxygen radicals; not reported long-term developmental and behavioral outcomes	-
Sildenafil	Neuroprotective agent; enhances hemodynamic redistribution and increases vascular density; improves microcirculation	The combined rate of death or survival with severe neurodevelopmental impairment was 57% at 18 months.
Metformin	A common treatment for diabetes type 2; antioxidant and anti-inflammatory agent; enhances neurogenesis; supports remyelination in neonatal white matter after injury	-
GCs	Effective for reducing inflammation; impacts brain development through intracellular glucocorticoid and mineralocorticoid receptors; administration to the neonatal brain confer neuroprotection and mitigate brain damage; increases blood pressure and reduces need for inotropes in neonates	Administration of GCs in preterm infants is a controversial topic.
Topiramate	Anticonvulsant drug; effective absorption, high bioavailability, good tolerability; considered as a neuroprotective drug; short-term use has minimal neurotoxic effects; low rate of seizures and no mortality events; reduction in the prevalence of epilepsy	The rate of death or severe neurological disability remains unchanged, but the percentage of adverse events has increased.
Magnesium sulphate	Ability to reduce pain and decrease the need for anesthetics; involves in vasodilatation, hemostasis and maintenance of the BBB; neuroprotective agent; supportive drug for newborns with HI injury; low rate of seizures and neurological problems	-
Sovateltide	Neuroprotective agent; potential drug for enhancing neuronal recovery after HIE; decreases oxidative stress and cell death in the neonatal HI; “first in class” drug for HIE	-
Cerebrolysin	Enhances the therapeutic efficacy and safety of thrombolytic agents; has complex mechanism of action, proven multimodal and pleiotropic effects; neuroprotection; long-term regeneration; improves social and speech composite; occurs minimal side effects	There are no changes either in the frequency or duration of the seizures.
NAC and calcitriol	Antioxidant and free radical scavenger (NAC); has a role in neuroplasticity, myelination and normal brain development (calcitriol); mitigates oxidative stress and contributes to neuroprotection of the injured neonatal brains; NAC increases GSH levels in brains after HIE	-
Vitamins and ibuprofen	Antioxidant agent (vitamin C); antioxidant and anti-inflammatory agent with neuroprotective properties (vitamin E); crucial for the development of the nervous system during embryonic development (vitamin E); anti-inflammatory drug that alleviates pain and reduces inflammation (ibuprofen)	The neuroprotective efficacy of vitamin E remains controversial due to its varying effectiveness. Observed incidence of mortality, neurological abnormalities, and developmental delay at 6 months of age.
Caffeine citrate	Has a neuroprotective effect; influences on neuronal functions	In animal models, it mitigates white matter brain damage. There is no data on the efficacy and safety of the therapy in newborns after hypoxic-ischemic (HI) injury. Some adverse events occur.
2-IB	Provides neuroprotection by inhibiting the neuronal and inducible forms of NOS; the aim of using 2-IB was to modulate the pathophysiological pathways activated by oxygen deprivation after HI	-
Citicoline	Aids in neurorepair; protects damaged tissue from mechanism of brain injury; enhances brain plasticity	-
RLS-0071	Anti-inflammatory agent, inhibits cellular and humoral inflammation; established as the therapy for HIE and neurotrophic pulmonary diseases	-
Xenon	Odorless gas use in inhalations; in neonates, it has a quick onset and provides anticonvulsant effects; provides most of neuroprotective benefits; reduces apoptotic cell death during phases of reperfusion injury; low rate of adverse events	There were a few deaths reported; TH with xenon was not associated with a reduction in mortality; there were no substantial differences between the groups in terms of the analyses of the selected biomarkers of brain damage or the occurrence of seizures during the initial hospitalization.

## Data Availability

No new data were created or analyzed in this study. Data sharing is not applicable to this article.

## References

[1] Pawale D., Fursule A., Tan J., Wagh D., Patole S., Rao S. (2024). Prevalence of Hearing Impairment in Neonatal Encephalopathy Due to Hypoxia-Ischemia: A Systematic Review and Meta-Analysis. Pediatr. Res..

[2] Allen K.A., Brandon D.H. (2011). Hypoxic Ischemic Encephalopathy: Pathophysiology and Experimental Treatments. Newborn Infant Nurs. Rev..

[3] Bruschettini M., Romantsik O., Moreira A., Ley D., Thébaud B. (2020). Stem Cell-based Interventions for the Prevention of Morbidity and Mortality Following Hypoxic-ischaemic Encephalopathy in Newborn Infants. Cochrane Database Syst. Rev..

[4] Vries L., Cowan F. (2009). Evolving Understanding of Hypoxic-Ischemic Encephalopathy in the Term Infant. Semin. Pediatr. Neurol..

[5] American College of Obstetricians and Gynecologists’ Task Force on Neonatal Encephalopathy (2014). Executive Summary: Neonatal Encephalopathy and Neurologic Outcome, Second Edition. Obstet. Gynecol..

[6] Cotten C.M., Shankaran S. (2010). Hypothermia for Hypoxic–Ischemic Encephalopathy. Expert Rev. Obstet. Gynecol..

[7] Gersh B.J., Stone G.W., White H.D., Holmes D.R. (2005). Pharmacological Facilitation of Primary Percutaneous Coronary Intervention for Acute Myocardial InfarctionIs the Slope of the Curve the Shape of the Future?. JAMA.

[8] Musiolik J., van Caster P., Skyschally A., Boengler K., Gres P., Schulz R., Heusch G. (2010). Reduction of Infarct Size by Gentle Reperfusion without Activation of Reperfusion Injury Salvage Kinases in Pigs. Cardiovasc. Res..

[9] Calcagno D.M., Zhang C., Toomu A., Huang K., Ninh V.K., Miyamoto S., Aguirre A.D., Fu Z., Heller Brown J., King K.R. (2021). SiglecF(HI) Marks Late-Stage Neutrophils of the Infarcted Heart: A Single-Cell Transcriptomic Analysis of Neutrophil Diversification. J. Am. Heart Assoc..

[10] Li J., Conrad C., Mills T.W., Berg N.K., Kim B., Ruan W., Lee J.W., Zhang X., Yuan X., Eltzschig H.K. (2021). PMN-Derived Netrin-1 Attenuates Cardiac Ischemia-Reperfusion Injury via Myeloid ADORA2B Signaling. J. Exp. Med..

[11] Fraccarollo D., Neuser J., Möller J., Riehle C., Galuppo P., Bauersachs J. (2021). Expansion of CD10neg Neutrophils and CD14+HLA-DRneg/Low Monocytes Driving Proinflammatory Responses in Patients with Acute Myocardial Infarction. eLife.

[12] Noc M., Laanmets P., Neskovic A., Petrović M., Stanetic B., Aradi D., Kiss R., Ungi I., Merkely B., Hudec M. A Multicentre, Prospective, Randomised Controlled Trial to Assess the Safety and Effectiveness of Cooling as an Adjunctive Therapy to Percutaneous Intervention in Patients with Acute Myocardial Infarction: The COOL AMI EU Pivotal Trial. https://eurointervention.pcronline.com/article/a-multicentre-prospective-randomised-controlled-trial-to-assess-the-safety-and-effectiveness-ofcooling-as-an-adjunctive-therapy-to-percutaneous-intervention-in-patients-with-acute-myocardial-infarction-thecoolamieu-pivotal-trial.

[13] Tissier R., Ghaleh B., Cohen M.V., Downey J.M., Berdeaux A. (2012). Myocardial Protection with Mild Hypothermia. Cardiovasc. Res..

[14] Heusch G. (2024). Myocardial Ischemia/Reperfusion: Translational Pathophysiology of Ischemic Heart Disease. Med.

[15] Ginet V., Pittet M.P., Rummel C., Osterheld M.C., Meuli R., Clarke P.G.H., Puyal J., Truttmann A.C. (2014). Dying Neurons in Thalamus of Asphyxiated Term Newborns and Rats Are Autophagic. Ann. Neurol..

[16] Ziemka-Nalecz M., Jaworska J., Sypecka J., Zalewska T. (2015). OGD Induced Modification of FAK- and PYK2-Coupled Pathways in Organotypic Hippocampal Slice Cultures. Brain Res..

[17] Ziemka-Nalecz M., Janowska J., Strojek L., Jaworska J., Zalewska T., Frontczak-Baniewicz M., Sypecka J. (2018). Impact of Neonatal Hypoxia-Ischaemia on Oligodendrocyte Survival, Maturation and Myelinating Potential. J. Cell. Mol. Med..

[18] Wootton S.H., Rysavy M., Davis P., Thio M., Romero-Lopez M., Holzapfel L.F., Thrasher T., Wade J.D., Owen L.S. (2024). Practical Approaches for Supporting Informed Consent in Neonatal Clinical Trials. Acta Paediatr..

[19] Janowska J., Gargas J., Zajdel K., Wieteska M., Lipinski K., Ziemka-Nalecz M., Frontczak-Baniewicz M., Sypecka J. (2024). Oligodendrocyte Progenitor Cells’ Fate after Neonatal Asphyxia—Puzzling Implications for the Development of Hypoxic–Ischemic Encephalopathy. Brain Pathol..

[20] Gargas J., Janowska J., Gebala P., Maksymiuk W., Sypecka J. (2024). Reactive Gliosis in Neonatal Disorders: Friend or Foe for Neuroregeneration?. Cells.

[21] Nagai A., Nakagawa E., Choi H.B., Hatori K., Kobayashi S., Kim S.U. (2001). Erythropoietin and Erythropoietin Receptors in Human CNS Neurons, Astrocytes, Microglia, and Oligodendrocytes Grown in Culture. J. Neuropathol. Exp. Neurol..

[22] Yamaji R., Okada T., Moriya M., Naito M., Tsuruo T., Miyatake K., Nakano Y. (1996). Brain Capillary Endothelial Cells Express Two Forms of Erythropoietin Receptor mRNA. Eur. J. Biochem..

[23] Yu X., Shacka J.J., Eells J.B., Suarez-Quian C., Przygodzki R.M., Beleslin-Cokic B., Lin C.-S., Nikodem V.M., Hempstead B., Flanders K.C. (2002). Erythropoietin Receptor Signalling Is Required for Normal Brain Development. Dev. Camb. Engl..

[24] Juul S.E., Yachnis A.T., Rojiani A.M., Christensen R.D. (1999). Immunohistochemical Localization of Erythropoietin and Its Receptor in the Developing Human Brain. Pediatr. Dev. Pathol..

[25] Gonzalez F.F., Larpthaveesarp A., McQuillen P., Derugin N., Wendland M., Spadafora R., Ferriero D.M. (2013). Erythropoietin Increases Neurogenesis and Oligodendrogliosis of SVZ Precursor Cells after Neonatal Stroke. Stroke J. Cereb. Circ..

[26] Kaneko N., Kako E., Sawamoto K. (2013). Enhancement of Ventricular-Subventricular Zone-Derived Neurogenesis and Oligodendrogenesis by Erythropoietin and Its Derivatives. Front. Cell. Neurosci..

[27] Xiong Y., Mahmood A., Meng Y., Zhang Y., Qu C., Schallert T., Chopp M. (2010). Delayed Administration of Erythropoietin Reduces Hippocampal Cell Loss, Enhances Angiogenesis and Neurogenesis, and Improves Functional Outcome Following Traumatic Brain Injury in Rats: Comparison of Treatment with Single Dose and Triple Dose. J. Neurosurg..

[28] Ikeda E. (2005). Cellular Response to Tissue Hypoxia and Its Involvement in Disease Progression. Pathol. Int..

[29] Villa P., van Beek J., Larsen A.K., Gerwien J., Christensen S., Cerami A., Brines M., Leist M., Ghezzi P., Torup L. (2007). Reduced Functional Deficits, Neuroinflammation, and Secondary Tissue Damage after Treatment of Stroke by Nonerythropoietic Erythropoietin Derivatives. J. Cereb. Blood Flow Metab..

[30] Juul S.E., Beyer R.P., Bammler T.K., McPherson R.J., Wilkerson J., Farin F.M. (2009). Microarray Analysis of High-Dose Recombinant Erythropoietin Treatment of Unilateral Brain Injury in Neonatal Mouse Hippocampus. Pediatr. Res..

[31] Wang L., Zhang Z.G., Zhang R.L., Gregg S.R., Hozeska-Solgot A., LeTourneau Y., Wang Y., Chopp M. (2006). Matrix Metalloproteinase 2 (MMP2) and MMP9 Secreted by Erythropoietin-Activated Endothelial Cells Promote Neural Progenitor Cell Migration. J. Neurosci..

[32] Wenger R.H. (2000). Mammalian Oxygen Sensing, Signalling and Gene Regulation. J. Exp. Biol..

[33] Maxwell P.H., Pugh C.W., Ratcliffe P.J. (1993). Inducible Operation of the Erythropoietin 3′ Enhancer in Multiple Cell Lines: Evidence for a Widespread Oxygen-Sensing Mechanism. Proc. Natl. Acad. Sci. USA.

[34] Jelkmann W. (2004). Molecular Biology of Erythropoietin. Intern. Med. Tokyo Jpn..

[35] Rankin E.B., Biju M.P., Liu Q., Unger T.L., Rha J., Johnson R.S., Simon M.C., Keith B., Haase V.H. (2007). Hypoxia-Inducible Factor–2 (HIF-2) Regulates Hepatic Erythropoietin in Vivo. J. Clin. Investig..

[36] Ratcliffe P.J. (2007). HIF-1 and HIF-2: Working Alone or Together in Hypoxia?. J. Clin. Investig..

[37] Jelkmann W., Lanfranco F., Strasburger C.J. (2016). Erythropoietin. Frontiers of Hormone Research.

[38] Rogers E.E., Bonifacio S.L., Glass H.C., Juul S.E., Chang T., Mayock D.E., Durand D.J., Song D., Barkovich A.J., Ballard R.A. (2014). Erythropoietin and Hypothermia for Hypoxic-Ischemic Encephalopathy. Pediatr. Neurol..

[39] Wu Y.W., Bauer L.A., Ballard R.A., Ferriero D.M., Glidden D.V., Mayock D.E., Chang T., Durand D.J., Song D., Bonifacio S.L. (2012). Erythropoietin for Neuroprotection in Neonatal Encephalopathy: Safety and Pharmacokinetics. Pediatrics.

[40] Massaro A.N., Wu Y.W., Bammler T.K., MacDonald J.W., Mathur A., Chang T., Mayock D., Mulkey S.B., van Meurs K., Afsharinejad Z. (2019). Dried Blood Spot Compared to Plasma Measurements of Blood-Based Biomarkers of Brain Injury in Neonatal Encephalopathy. Pediatr. Res..

[41] Wu Y.W., Goodman A.M., Chang T., Mulkey S.B., Gonzalez F.F., Mayock D.E., Juul S.E., Mathur A.M., Van Meurs K., McKinstry R.C. (2020). Placental Pathology and Neonatal Brain MRI in a Randomized Trial of Erythropoietin for Hypoxic–Ischemic Encephalopathy. Pediatr. Res..

[42] Mulkey S.B., Ramakrishnaiah R.H., McKinstry R.C., Chang T., Mathur A.M., Mayock D.E., Meurs K.P.V., Schaefer G.B., Luo C., Bai S. (2017). Erythropoietin and Brain Magnetic Resonance Imaging Findings in Hypoxic-Ischemic Encephalopathy: Volume of Acute Brain Injury and 1-Year Neurodevelopmental Outcome. J. Pediatr..

[43] Elmahdy H., El-Mashad A.-R., El-Bahrawy H., El-Gohary T., El-Barbary A., Aly H. (2010). Human Recombinant Erythropoietin in Asphyxia Neonatorum: Pilot Trial. Pediatrics.

[44] Baserga M.C., Beachy J.C., Roberts J.K., Ward R.M., DiGeronimo R.J., Walsh W.F., Ohls R.K., Anderson J., Mayock D.E., Juul S.E. (2015). Darbepoetin Administration to Neonates Undergoing Cooling for Encephalopathy: A Safety and Pharmacokinetic Trial. Pediatr. Res..

[45] Wu Y.W., Comstock B.A., Gonzalez F.F., Mayock D.E., Goodman A.M., Maitre N.L., Chang T., Van Meurs K.P., Lampland A.L., Bendel-Stenzel E. (2022). Trial of Erythropoietin for Hypoxic–Ischemic Encephalopathy in Newborns. N. Engl. J. Med..

[46] Reiter R.J., Tan D.X., Galano A. (2014). Melatonin: Exceeding Expectations. Physiology.

[47] Kennaway D.J. (2020). Measuring Melatonin by Immunoassay. J. Pineal Res..

[48] Cecon E., Boutin J.A., Jockers R. (2023). Molecular Characterization and Pharmacology of Melatonin Receptors in Animals. Receptors.

[49] Reiter R.J., Rosales-Corral S., Tan D.X., Jou M.J., Galano A., Xu B. (2017). Melatonin as a Mitochondria-Targeted Antioxidant: One of Evolution’s Best Ideas. Cell. Mol. Life Sci. CMLS.

[50] Hardeland R., Cardinali D.P., Brown G.M., Pandi-Perumal S.R. (2015). Melatonin and Brain Inflammaging. Prog. Neurobiol..

[51] Aly H., Elmahdy H., El-Dib M., Rowisha M., Awny M., El-Gohary T., Elbatch M., Hamisa M., El-Mashad A.-R. (2015). Melatonin Use for Neuroprotection in Perinatal Asphyxia: A Randomized Controlled Pilot Study. J. Perinatol..

[52] Maiwald C.A., Annink K.V., Rüdiger M., Benders M.J.N.L., van Bel F., Allegaert K., Naulaers G., Bassler D., Klebermaß-Schrehof K., Vento M. (2019). Effect of Allopurinol in Addition to Hypothermia Treatment in Neonates for Hypoxic-Ischemic Brain Injury on Neurocognitive Outcome (ALBINO): Study Protocol of a Blinded Randomized Placebo-Controlled Parallel Group Multicenter Trial for Superiority (Phase III). BMC Pediatr..

[53] Deferm N., Annink K.V., Faelens R., Schroth M., Maiwald C.A., el Bakkali L., van Bel F., Benders M.J.N.L., van Weissenbruch M.M., Hagen A. (2021). Glomerular Filtration Rate in Asphyxiated Neonates Under Therapeutic Whole-Body Hypothermia, Quantified by Mannitol Clearance. Clin. Pharmacokinet..

[54] Engel C., Rüdiger M., Benders M.J.N.L., van Bel F., Allegaert K., Naulaers G., Bassler D., Klebermaß-Schrehof K., Vento M., Vilan A. (2024). Detailed Statistical Analysis Plan for ALBINO: Effect of Allopurinol in Addition to Hypothermia for Hypoxic-Ischemic Brain Injury on Neurocognitive Outcome—A Blinded Randomized Placebo-Controlled Parallel Group Multicenter Trial for Superiority (Phase III). Trials.

[55] Chu W.Y., Annink K.V., Nijstad A.L., Maiwald C.A., Schroth M., el Bakkali L., van Bel F., Benders M.J.N.L., van Weissenbruch M.M., Hagen A. (2022). Pharmacokinetic/Pharmacodynamic Modelling of Allopurinol, Its Active Metabolite Oxypurinol, and Biomarkers Hypoxanthine, Xanthine and Uric Acid in Hypoxic-Ischemic Encephalopathy Neonates. Clin. Pharmacokinet..

[56] Klumper J., Kaandorp J.J., Schuit E., Groenendaal F., Koopman-Esseboom C., Mulder E.J.H., Van Bel F., Benders M.J.N.L., Mol B.W.J., van Elburg R.M. (2018). Behavioral and Neurodevelopmental Outcome of Children after Maternal Allopurinol Administration during Suspected Fetal Hypoxia: 5-Year Follow up of the ALLO-Trial. PLoS ONE.

[57] Kaandorp J.J., Benders M.J.N.L., Schuit E., Rademaker C.M.A., Oudijk M.A., Porath M.M., Oetomo S.B., Wouters M.G.A.J., van Elburg R.M., Franssen M.T.M. (2015). Maternal Allopurinol Administration during Suspected Fetal Hypoxia: A Novel Neuroprotective Intervention? A Multicentre Randomised Placebo Controlled Trial. Arch. Dis. Child.-Fetal Neonatal Ed..

[58] Kaandorp J.J., van den Broek M.P.H., Benders M.J.N.L., Oudijk M.A., Porath M.M., Oetomo S.B., Wouters M.G.a.J., van Elburg R., Franssen M.T.M., Bos A.F. (2014). Rapid Target Allopurinol Concentrations in the Hypoxic Fetus after Maternal Administration during Labour. Arch. Dis. Child.-Fetal Neonatal Ed..

[59] Torrance H.L., Benders M.J., Derks J.B., Rademaker C.M.A., Bos A.F., Van Den Berg P., Longini M., Buonocore G., Venegas M., Baquero H. (2009). Maternal Allopurinol During Fetal Hypoxia Lowers Cord Blood Levels of the Brain Injury Marker S-100B. Pediatrics.

[60] Yamamoto T., Moriwaki Y., Suda M., Nasako Y., Takahashi S., Hiroishi K., Nakano T., Hada T., Higashino K. (1993). Effect of BOF-4272 on the Oxidation of Allopurinol and Pyrazinamide in Vivo. Biochem. Pharmacol..

[61] McCord J.M. (1985). Oxygen-Derived Free Radicals in Postischemic Tissue Injury. N. Engl. J. Med..

[62] van Bel F., Groenendaal F. (2016). Drugs for Neuroprotection after Birth Asphyxia: Pharmacologic Adjuncts to Hypothermia. Semin. Perinatol..

[63] Annink K.V., Franz A.R., Derks J.B., Rudiger M., van Bel F., Benders M.J.N.L. (2017). Allopurinol: Old Drug, New Indication in Neonates?. Curr. Pharm. Des..

[64] Archer S.L., Huang J.M., Hampl V., Nelson D.P., Shultz P.J., Weir E.K. (1994). Nitric Oxide and cGMP Cause Vasorelaxation by Activation of a Charybdotoxin-Sensitive K Channel by cGMP-Dependent Protein Kinase. Proc. Natl. Acad. Sci. USA.

[65] Perez K.M., Laughon M. (2015). Sildenafil in Term and Premature Infants: A Systematic Review. Clin. Ther..

[66] Ölmestig J.N.E., Marlet I.R., Hainsworth A.H., Kruuse C. (2017). Phosphodiesterase 5 Inhibition as a Therapeutic Target for Ischemic Stroke: A Systematic Review of Preclinical Studies. Cell. Signal..

[67] Zhang R., Wang Y., Zhang L., Zhang Z., Tsang W., Lu M., Zhang L., Chopp M. (2002). Sildenafil (Viagra) Induces Neurogenesis and Promotes Functional Recovery After Stroke in Rats. Stroke.

[68] Charriaut-Marlangue C., Nguyen T., Bonnin P., Duy A.P., Leger P.-L., Csaba Z., Pansiot J., Bourgeois T., Renolleau S., Baud O. (2014). Sildenafil Mediates Blood-Flow Redistribution and Neuroprotection After Neonatal Hypoxia-Ischemia. Stroke.

[69] Moretti R., Leger P.-L., Besson V.C., Csaba Z., Pansiot J., Di Criscio L., Gentili A., Titomanlio L., Bonnin P., Baud O. (2016). Sildenafil, a Cyclic GMP Phosphodiesterase Inhibitor, Induces Microglial Modulation after Focal Ischemia in the Neonatal Mouse Brain. J. Neuroinflamm..

[70] Li L., Jiang Q., Zhang L., Ding G., Zhang Z.G., Li Q., Ewing J.R., Lu M., Panda S., Ledbetter K.A. (2007). Angiogenesis and Improved Cerebral Blood Flow in the Ischemic Boundary Area Detected by MRI after Administration of Sildenafil to Rats with Embolic Stroke. Brain Res..

[71] Zinni M., Pansiot J., Léger P.-L., El Kamouh M., Baud O. (2021). Sildenafil-Mediated Neuroprotection from Adult to Neonatal Brain Injury: Evidence, Mechanisms, and Future Translation. Cells.

[72] Julien P., Zinni M., Bonnel N., El Kamouh M., Odorcyk F., Peters L., Gautier E.-F., Leduc M., Broussard C., Baud O. (2024). Synergistic Effect of Sildenafil Combined with Controlled Hypothermia to Alleviate Microglial Activation after Neonatal Hypoxia–Ischemia in Rats. J. Neuroinflamm..

[73] Wintermark P., Lapointe A., Steinhorn R., Rampakakis E., Burhenne J., Meid A.D., Bajraktari-Sylejmani G., Khairy M., Altit G., Adamo M.-T. (2024). Feasibility and Safety of Sildenafil to Repair Brain Injury Secondary to Birth Asphyxia (SANE-01): A Randomized, Double-Blind, Placebo-Controlled Phase Ib Clinical Trial. J. Pediatr..

[74] Zhou J., Massey S., Story D., Li L. (2018). Metformin: An Old Drug with New Applications. Int. J. Mol. Sci..

[75] Venna V.R., Li J., Hammond M.D., Mancini N.S., McCullough L.D. (2014). Chronic Metformin Treatment Improves Post-Stroke Angiogenesis and Recovery after Experimental Stroke. Eur. J. Neurosci..

[76] Wang Y.-W., He S.-J., Feng X., Cheng J., Luo Y.-T., Tian L., Huang Q. (2017). Metformin: A Review of Its Potential Indications. Drug Des. Devel. Ther..

[77] Ruan C., Guo H., Gao J., Wang Y., Liu Z., Yan J., Li X., Lv H. (2021). Neuroprotective Effects of Metformin on Cerebral Ischemia-reperfusion Injury by Regulating PI3K/Akt Pathway. Brain Behav..

[78] Sharma S., Nozohouri S., Vaidya B., Abbruscato T. (2021). Repurposing Metformin to Treat Age-Related Neurodegenerative Disorders and Ischemic Stroke. Life Sci..

[79] Fang M., Jiang H., Ye L., Cai C., Hu Y., Pan S., Li P., Xiao J., Lin Z. (2017). Metformin Treatment after the Hypoxia-Ischemia Attenuates Brain Injury in Newborn Rats. Oncotarget.

[80] Study Details|Metformin Treatment in Infants After Perinatal Brain Injury|ClinicalTrials.Gov. https://www.clinicaltrials.gov/study/NCT05590676?cond=neonatal%20hypoxia-ischemia&rank=81.

[81] Kovacs K., Szakmar E., Meder U., Szakacs L., Cseko A., Vatai B., Szabo A.J., McNamara P.J., Szabo M., Jermendy A. (2019). A Randomized Controlled Study of Low-Dose Hydrocortisone Versus Placebo in Dopamine-Treated Hypotensive Neonates Undergoing Hypothermia Treatment for Hypoxic−Ischemic Encephalopathy. J. Pediatr..

[82] Filippi L., Fiorini P., Daniotti M., Catarzi S., Savelli S., Fonda C., Bartalena L., Boldrini A., Giampietri M., Scaramuzzo R. (2012). Safety and Efficacy of Topiramate in Neonates with Hypoxic Ischemic Encephalopathy Treated with Hypothermia (NeoNATI). BMC Pediatr..

[83] Filippi L., Fiorini P., Catarzi S., Berti E., Padrini L., Landucci E., Donzelli G., Bartalena L., Fiorentini E., Boldrini A. (2018). Safety and Efficacy of Topiramate in Neonates with Hypoxic Ischemic Encephalopathy Treated with Hypothermia (NeoNATI): A Feasibility Study. J. Matern. Fetal Neonatal Med..

[84] Siddiqui M.A., Butt T.K. (2021). Role of Intravenous Magnesium Sulphate in Term Neonates with Hypoxic Ischemic Encephalopathy (HIE) in a Low-Income Country: A Randomised Clinical Trial. J. Coll. Physicians Surg. Pak. JCPSP.

[85] Hassanein S.M.A., Deifalla S.M., El-Houssinie M., Mokbel S.A. (2016). Safety and Efficacy of Cerebrolysin in Infants with Communication Defects Due to Severe Perinatal Brain Insult: A Randomized Controlled Clinical Trial. J. Clin. Neurol..

[86] Moss H.G., Brown T.R., Wiest D.B., Jenkins D.D. (2018). N-Acetylcysteine Rapidly Replenishes Central Nervous System Glutathione Measured via Magnetic Resonance Spectroscopy in Human Neonates with Hypoxic-Ischemic Encephalopathy. J. Cereb. Blood Flow Metab..

[87] Sánchez-Illana Á., Thayyil S., Montaldo P., Jenkins D., Quintás G., Oger C., Galano J.-M., Vigor C., Durand T., Vento M. (2017). Novel Free-Radical Mediated Lipid Peroxidation Biomarkers in Newborn Plasma. Anal. Chim. Acta.

[88] Aly H., Abd-Rabboh L., El-Dib M., Nawwar F., Hassan H., Aaref M., Abdelrahman S., Elsayed A. (2009). Ascorbic Acid Combined with Ibuprofen in Hypoxic Ischemic Encephalopathy: A Randomized Controlled Trial. J. Perinatol..

[89] Jackson W., Gonzalez D., Greenberg R.G., Lee Y.Z., Laughon M.M. (2024). A Phase I Trial of Caffeine to Evaluate Safety in Infants with Hypoxic-Ischemic Encephalopathy. J. Perinatol..

[90] Azzopardi D., Robertson N.J., Bainbridge A., Cady E., Charles-Edwards G., Deierl A., Fagiolo G., Franks N.P., Griffiths J., Hajnal J. (2016). Moderate Hypothermia within 6 h of Birth plus Inhaled Xenon versus Moderate Hypothermia Alone after Birth Asphyxia (TOBY-Xe): A Proof-of-Concept, Open-Label, Randomised Controlled Trial. Lancet Neurol..

[91] Dingley J., Liu X., Gill H., Smit E., Sabir H., Tooley J., Chakkarapani E., Windsor D., Thoresen M. (2015). The Feasibility of Using a Portable Xenon Delivery Device to Permit Earlier Xenon Ventilation with Therapeutic Cooling of Neonates during Ambulance Retrieval. Anesth. Analg..

[92] Patra A., Huang H., Bauer J.A., Giannone P.J. (2017). Neurological Consequences of Systemic Inflammation in the Premature Neonate. Neural Regen. Res..

[93] Gallini F., Coppola M., De Rose D.U., Maggio L., Arena R., Romano V., Cota F., Ricci D., Romeo D.M., Mercuri E.M. (2021). Neurodevelopmental Outcomes in Very Preterm Infants: The Role of Severity of Bronchopulmonary Dysplasia. Early Hum. Dev..

[94] Harding B., Conception K., Li Y., Zhang L. (2016). Glucocorticoids Protect Neonatal Rat Brain in Model of Hypoxic-Ischemic Encephalopathy (HIE). Int. J. Mol. Sci..

[95] Guerrini R., Parmeggiani L. (2006). Topiramate and Its Clinical Applications in Epilepsy. Expert Opin. Pharmacother..

[96] Shank R.P., Gardocki J.F., Streeter A.J., Maryanoff B.E. (2000). An Overview of the Preclinical Aspects of Topiramate: Pharmacology, Pharmacokinetics, and Mechanism of Action. Epilepsia.

[97] Filippi L., Poggi C., la Marca G., Furlanetto S., Fiorini P., Cavallaro G., Plantulli A., Donzelli G., Guerrini R. (2010). Oral Topiramate in Neonates with Hypoxic Ischemic Encephalopathy Treated with Hypothermia: A Safety Study. J. Pediatr..

[98] Glier C., Dzietko M., Bittigau P., Jarosz B., Korobowicz E., Ikonomidou C. (2004). Therapeutic Doses of Topiramate Are Not Toxic to the Developing Rat Brain. Exp. Neurol..

[99] Noh M.-R., Kim S.K., Sun W., Park S.K., Choi H.C., Lim J.H., Kim I.H., Kim H.-J., Kim H., Eun B.-L. (2006). Neuroprotective Effect of Topiramate on Hypoxic Ischemic Brain Injury in Neonatal Rats. Exp. Neurol..

[100] Albrecht E., Kirkham K.R., Liu S.S., Brull R. (2013). Peri-Operative Intravenous Administration of Magnesium Sulphate and Postoperative Pain: A Meta-Analysis. Anaesthesia.

[101] Do S.-H. (2013). Magnesium: A Versatile Drug for Anesthesiologists. Korean J. Anesthesiol..

[102] Sohn H.-M., Jheon S.-H., Nam S., Do S.-H. (2017). Magnesium Sulphate Improves Pulmonary Function after Video-Assisted Thoracoscopic Surgery: A Randomised Double-Blind Placebo-Controlled Study. Eur. J. Anaesthesiol. EJA.

[103] Newcomer J.W., Farber N.B., Olney J.W. (2000). NMDA Receptor Function, Memory, and Brain Aging. Dialogues Clin. Neurosci..

[104] Kemp P.A., Gardiner S.M., March J.E., Rubin P.C., Bennett T. (1999). Assessment of the Effects of Endothelin-1 and Magnesium Sulphate on Regional Blood Flows in Conscious Rats, by the Coloured Microsphere Reference Technique. Br. J. Pharmacol..

[105] Chang J.J., Mack W.J., Saver J.L., Sanossian N. (2014). Magnesium: Potential Roles in Neurovascular Disease. Front. Neurol..

[106] Shea K.L., Palanisamy A. (2015). What Can You Do to Protect the Newborn Brain?. Curr. Opin. Anesthesiol..

[107] Yanagisawa M., Kurihara H., Kimura S., Tomobe Y., Kobayashi M., Mitsui Y., Yazaki Y., Goto K., Masaki T. (1988). A Novel Potent Vasoconstrictor Peptide Produced by Vascular Endothelial Cells. Nature.

[108] Ramos M.D., Briyal S., Prazad P., Gulati A. (2022). Neuroprotective Effect of Sovateltide (IRL 1620, PMZ 1620) in a Neonatal Rat Model of Hypoxic-Ischemic Encephalopathy. Neuroscience.

[109] Ranjan A.K., Gulati A. (2023). Advances in Therapies to Treat Neonatal Hypoxic-Ischemic Encephalopathy. J. Clin. Med..

[110] Teng H., Li C., Zhang Y., Lu M., Chopp M., Zhang Z.G., Melcher-Mourgas M., Fleckenstein B. (2021). Therapeutic Effect of Cerebrolysin on Reducing Impaired Cerebral Endothelial Cell Permeability. NeuroReport.

[111] Muresanu D.F., Strilciuc S., Stan A. (2019). Current Drug Treatment of Acute Ischemic Stroke: Challenges and Opportunities. CNS Drugs.

[112] Samuni Y., Goldstein S., Dean O.M., Berk M. (2013). The Chemistry and Biological Activities of N-Acetylcysteine. Biochim. Biophys. Acta BBA-Gen. Subj..

[113] Frye R.E., Andrus J.P., Lemley K.V., De Rosa S.C., Ghezzi P., Holmgren A., Jones D., Jahoor F., Kopke R., Cotgreave I., Frye R.E., Berk M. (2019). Pharmacology, Formulations, and Adverse Effects. The Therapeutic Use of N-Acetylcysteine (NAC) in Medicine.

[114] Arakawa M., Ito Y. (2007). N-Acetylcysteine and Neurodegenerative Diseases: Basic and Clinical Pharmacology. Cerebellum Lond. Engl..

[115] Jenkins D.D., Moss H.G., Brown T.R., Yazdani M., Thayyil S., Montaldo P., Vento M., Kuligowski J., Wagner C., Hollis B.W. (2021). NAC and Vitamin D Improve CNS and Plasma Oxidative Stress in Neonatal HIE and Are Associated with Favorable Long-Term Outcomes. Antioxidants.

[116] Cui X., McGrath J.J., Burne T.H.J., Mackay-Sim A., Eyles D.W. (2007). Maternal Vitamin D Depletion Alters Neurogenesis in the Developing Rat Brain. Int. J. Dev. Neurosci..

[117] Eyles D., Almeras L., Benech P., Patatian A., Mackay-Sim A., McGrath J., Féron F. (2007). Developmental Vitamin D Deficiency Alters the Expression of Genes Encoding Mitochondrial, Cytoskeletal and Synaptic Proteins in the Adult Rat Brain. J. Steroid Biochem. Mol. Biol..

[118] Gomez-Pinedo U., Cuevas J.A., Benito-Martín M.S., Moreno-Jiménez L., Esteban-Garcia N., Torre-Fuentes L., Matías-Guiu J.A., Pytel V., Montero P., Matías-Guiu J. (2019). Vitamin D Increases Remyelination by Promoting Oligodendrocyte Lineage Differentiation. Brain Behav..

[119] Al-Niaimi F., Chiang N.Y.Z. (2017). Topical Vitamin C and the Skin: Mechanisms of Action and Clinical Applications. J. Clin. Aesthetic Dermatol..

[120] Mohd Zaffarin A.S., Ng S.-F., Ng M.H., Hassan H., Alias E. (2020). Pharmacology and Pharmacokinetics of Vitamin E: Nanoformulations to Enhance Bioavailability. Int. J. Nanomed..

[121] La Torre M.E., Villano I., Monda M., Messina A., Cibelli G., Valenzano A., Pisanelli D., Panaro M.A., Tartaglia N., Ambrosi A. (2021). Role of Vitamin E and the Orexin System in Neuroprotection. Brain Sci..

[122] Singhi S., Johnston M. (2019). Recent Advances in Perinatal Neuroprotection. F1000Research.

[123] Traber M.G. (2021). Vitamin E: Necessary Nutrient for Neural Development and Cognitive Function. Proc. Nutr. Soc..

[124] Brion L.P., Bell E.F., Raghuveer T.S. (2003). Vitamin E Supplementation for Prevention of Morbidity and Mortality in Preterm Infants. Cochrane Database Syst. Rev..

[125] Bell E.F., Hansen N.I., Brion L.P., Ehrenkranz R.A., Kennedy K.A., Walsh M.C., Shankaran S., Acarregui M.J., Johnson K.J., Hale E.C. (2013). Serum Tocopherol Levels in Very Preterm Infants After a Single Dose of Vitamin E at Birth. Pediatrics.

[126] Paul I.M., Walson P.D. (2021). Acetaminophen and Ibuprofen in the Treatment of Pediatric Fever: A Narrative Review. Curr. Med. Res. Opin..

[127] Janitschke D., Lauer A.A., Bachmann C.M., Seyfried M., Grimm H.S., Hartmann T., Grimm M.O.W. (2020). Unique Role of Caffeine Compared to Other Methylxanthines (Theobromine, Theophylline, Pentoxifylline, Propentofylline) in Regulation of AD Relevant Genes in Neuroblastoma SH-SY5Y Wild Type Cells. Int. J. Mol. Sci..

[128] Fredholm B.B., Bättig K., Holmén J., Nehlig A., Zvartau E.E. (1999). Actions of Caffeine in the Brain with Special Reference to Factors That Contribute to Its Widespread Use. Pharmacol. Rev..

[129] Fredholm B.B., Chen J.-F., Cunha R.A., Svenningsson P., Vaugeois J.-M. (2005). Adenosine and Brain Function. International Review of Neurobiology.

[130] Lopes J.P., Pliássova A., Cunha R.A. (2019). The Physiological Effects of Caffeine on Synaptic Transmission and Plasticity in the Mouse Hippocampus Selectively Depend on Adenosine A1 and A2A Receptors. Biochem. Pharmacol..

[131] Cunha R.A. (2016). How Does Adenosine Control Neuronal Dysfunction and Neurodegeneration?. J. Neurochem..

[132] Yang L., Yu X., Zhang Y., Liu N., Xue X., Fu J. (2022). Caffeine Treatment Started before Injury Reduces Hypoxic–Ischemic White-Matter Damage in Neonatal Rats by Regulating Phenotypic Microglia Polarization. Pediatr. Res..

[133] Dobson N.R., Hunt C.E. (2013). Pharmacology Review: Caffeine Use in Neonates: Indications, Pharmacokinetics, Clinical Effects, Outcomes. NeoReviews.

[134] Sup S.J., Green B.G., Grant S.K. (1994). 2-Iminobiotin Is an Inhibitor of Nitric Oxide Synthases. Biochem. Biophys. Res. Commun..

[135] Fan X., van Bel F. (2010). Pharmacological Neuroprotection after Perinatal Asphyxia. J. Matern. Fetal Neonatal Med..

[136] Favié L.M.A., Cox A.R., van den Hoogen A., Nijboer C.H.A., Peeters-Scholte C.M.P.C.D., van Bel F., Egberts T.C.G., Rademaker C.M.A., Groenendaal F. (2018). Nitric Oxide Synthase Inhibition as a Neuroprotective Strategy Following Hypoxic–Ischemic Encephalopathy: Evidence From Animal Studies. Front. Neurol..

[137] van Hoogdalem E.-J., Peeters-Scholte C.M.P.C.D., Leufkens P.W.T.J., Hartstra J., van Lier J.J., de Leede L.G.J. (2020). First-in-Human Study of the Safety, Tolerability, Pharmacokinetics and -Preliminary Dynamics of Neuroprotectant 2-Iminobiotin in Healthy Subjects. Curr. Clin. Pharmacol..

[138] Iulia C., Ruxandra T., Costin L.-B., Liliana-Mary V. (2017). Citicoline—A Neuroprotector with Proven Effects on Glaucomatous Disease. Romanian J. Ophthalmol..

[139] Salamah A., Mehrez M., Faheem A., El Amrousy D. (2021). Efficacy of Citicoline as a Neuroprotector in Children with Post Cardiac Arrest: A Randomized Controlled Clinical Trial. Eur. J. Pediatr..

[140] Hobson A., Baines J., Weiss M.D. (2013). Beyond Hypothermia: Alternative Therapies for Hypoxic Ischemic Encephalopathy. Open Pharmacol. J..

[141] Álvarez-Sabín J., Román G.C. (2013). The Role of Citicoline in Neuroprotection and Neurorepair in Ischemic Stroke. Brain Sci..

[142] Hair P.S., Enos A.I., Krishna N.K., Cunnion K.M. (2018). Inhibition of Immune Complex Complement Activation and Neutrophil Extracellular Trap Formation by Peptide Inhibitor of Complement C1. Front. Immunol..

[143] Goss J., Hair P., Kumar P., Iacono G., Redden L., Morelli G., Krishna N., Thienel U., Cunnion K. (2023). RLS-0071, a Dual-Targeting Anti-Inflammatory Peptide–Biomarker Findings from a First in Human Clinical Trial. Transl. Med. Commun..

[144] Rüegger C.M., Davis P.G., Cheong J.L. (2018). Xenon as an Adjuvant to Therapeutic Hypothermia in Near-term and Term Newborns with Hypoxic-ischaemic Encephalopathy. Cochrane Database Syst. Rev..

[145] Chakkarapani E., Dingley J., Liu X., Hoque N., Aquilina K., Porter H., Thoresen M. (2010). Xenon Enhances Hypothermic Neuroprotection in Asphyxiated Newborn Pigs. Ann. Neurol..

[146] Dingley J., Hobbs C., Ferguson J., Stone J., Thoresen M. (2008). Xenon/Hypothermia Neuroprotection Regimes in Spontaneously Breathing Neonatal Rats After Hypoxic-Ischemic Insult: The Respiratory and Sedative Effects. Anesth. Analg..

[147] Thoresen M., Hobbs C.E., Wood T., Chakkarapani E., Dingley J. (2009). Cooling Combined with Immediate or Delayed Xenon Inhalation Provides Equivalent Long-Term Neuroprotection after Neonatal Hypoxia—Ischemia. J. Cereb. Blood Flow Metab..

[148] Franks N.P., Dickinson R., de Sousa S.L.M., Hall A.C., Lieb W.R. (1998). How Does Xenon Produce Anaesthesia?. Nature.

[149] Armstrong S.P., Banks P.J., McKitrick T.J.W., Geldart C.H., Edge C.J., Babla R., Simillis C., Franks N.P., Dickinson R. (2012). Identification of Two Mutations (F758W and F758Y) in the N -Methyl-D-Aspartate Receptor Glycine-Binding Site That Selectively Prevent Competitive Inhibition by Xenon without Affecting Glycine Binding. Anesthesiology.

[150] Dickinson R., Peterson B.K., Banks P., Simillis C., Martin J.C.S., Valenzuela C.A., Maze M., Franks N.P. (2007). Competitive Inhibition at the Glycine Site of the N -Methyl-d-Aspartate Receptor by the Anesthetics Xenon and Isoflurane: Evidence from Molecular Modeling and Electrophysiology. Anesthesiology.

[151] Ma D., Lim T., Xu J., Tang H., Wan Y., Zhao H., Hossain M., Maxwell P.H., Maze M. (2009). Xenon Preconditioning Protects against Renal Ischemic-Reperfusion Injury via HIF-1α Activation. J. Am. Soc. Nephrol. JASN.

[152] Bauchner H., Rivara F.P. (2023). Improving Child Health Research: The Role of Randomized Clinical Trials. J. Pediatr..

[153] Marques K.L., Rodrigues V., Balduci C.T.N., Montes G.C., Barradas P.C., Cunha-Rodrigues M.C. (2024). Emerging Therapeutic Strategies in Hypoxic-Ischemic Encephalopathy: A Focus on Cognitive Outcomes. Front. Pharmacol..

[154] Gulati A., Agrawal N., Vibha D., Misra U.K., Paul B., Jain D., Pandian J., Borgohain R. (2021). Safety and Efficacy of Sovateltide (IRL-1620) in a Multicenter Randomized Controlled Clinical Trial in Patients with Acute Cerebral Ischemic Stroke. CNS Drugs.

[155] Rüegger C.M., Dawson J.A., Donath S.M., Owen L.S., Davis P.G. (2017). Nonpublication and Discontinuation of Randomised Controlled Trials in Newborns. Acta Paediatr..

